# Bacterial lipid synthesizing enzymes PlsY and PlsC utilize both stereo-forms of glycerol-phosphate

**DOI:** 10.1038/s44319-026-00827-z

**Published:** 2026-06-12

**Authors:** Philipp Rieche, Mirthe Hoekzema, Sergiy Gan, Greetje A Berrelkamp-Lahpor, Adanna W Ezissi, Marten Exterkate, Arnold J M Driessen

**Affiliations:** 1 Membrane Biogenesis and Lipidomics, Institute of Biochemistry, Heinrich Heine University, Düsseldorf, Germany; 2 Department of Molecular Microbiology, Groningen Biomolecular Sciences and Bio- technology Institute, University of Groningen, Groningen, The Netherlands

**Keywords:** Evolution & Ecology, Membranes & Trafficking

## Abstract

The chemical composition of membrane lipids differs between eukarya, bacteria and archaea. The central dogma posits that the stereochemistry of phospholipids in bacteria is distinct from archaea. Bacterial phospholipids consist of fatty acid lipid tails esterified to the *sn*-glycerol 3-phosphate lipid backbone (G3P), whereas archaeal phospholipids comprise isoprenoid lipid tails ether-linked to the stereochemical different *sn*-glycerol 1-phosphate (G1P). This segregation, the “lipid divide”, is however not as strict as previously thought. Recent reports demonstrate that both glycerol-phosphate backbones are present in phospholipids from various Gram-positive bacteria. To test if the stereochemical variability can be attributed to conventional lipid biosynthesis, we characterize the stereospecificity of the relevant glycerol-phosphate acyltransferases PlsY and PlsB, as well as the lysophosphatidic acid acyltransferase PlsC, catalyzing the key steps in phospholipid biosynthesis yielding phosphatidic acid, both in the Gram-positive *B. subtilis* and the Gram-negative *E. coli*. While PlsB is strictly stereospecific for glycerol 3-phosphate, PlsY and PlsC can utilize both stereo-forms of the glycerol-phosphate. Hence, the variability in lipid backbone stereochemistry is an intrinsic part of bacterial phospholipid biogenesis, questioning the supposedly strict stereochemical segregation of bacteria and archaea after the lipid divide.

## Synopsis

The bacterial acyltransferases PlsY and PlsC can utilize both the “bacterial” and “archaeal” glycerol-phosphate stereo-forms, suggesting that backbone variability is an intrinsic feature of bacterial membrane phospholipid biogenesis rather than an anomaly.

- Present ubiquitously in bacteria, the acyltransferases PlsY and PlsC utilize both stereo-forms of glycerol-phosphate.
- The acyltranferase PlsB is stereospecific for glycerol-3-phosphate.
- Backbone heterochirality of phospholipids observed in some bacteria can be attributed to PlsY activity.

## Abstract (web summary)

The bacterial acyltransferases PlsY and PlsC can utilize both, the “bacterial” and “archaeal” glycerol-phosphate stereo-forms, suggesting that backbone variability is an intrinsic feature of bacterial membrane phospholipid biogenesis rather than an anomaly.

## Graphical abstract

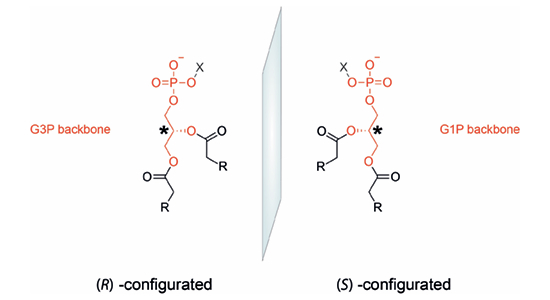

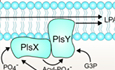

## Introduction

Phospholipids are the primary constituents of cellular membranes, playing a crucial role in preserving the integrity of living cells. Despite the structural similarities, the chemical composition of typical membrane phospholipids differs throughout the three domains of life. Archaeal phospholipids consist of a *sn*-glycerol-1-phosphate (G1P) backbone (*S-*configuration) ether-linked to isoprenoid chains. In contrast typical bacterial/eukaryotic phospholipids consist of fatty acids that are ester-linked to *sn*-glycerol-3-phosphate (G3P) (*R*-configuration). This division in membrane phospholipid composition is commonly referred to as the lipid divide, and is thought to have occurred in early evolution, when archaea and bacteria diverged from the last universal common ancestor (LUCA) (Koga and Morii, 2007). Despite decades of research, the lipid membrane composition of LUCA, as well as the cause of the lipid divide, remain unsolved. A prevalent theory assumes the existence of a pre-cell possessing a heterochiral mixed membrane, that as a consequence of evolutionary adaption and selective advantage diverged into subpopulations with homochiral membranes containing either ether- or ester-bonded phospholipids, increasing membrane stability (Wachtershauser, 2003; Lombard et al, 2012). The divide seems evident from the genetic background of the dehydrogenase superfamily, responsible for the synthesis of the glycerol-phosphate lipid backbone. The dehydrogenases in archaea and bacteria are structurally unrelated, yielding G1P or G3P, respectively (Martin et al, 2021). Hence, these enzymes must have evolved independently in bacteria and archaea (Pereto et al, 2004).

Advanced phylogenetic analysis of various archaea could show that some classes of *Euryarchaeota* (*e.g*., *Halobacteria* and *Methanomicrobia)* but also some *Crenarchaeota* (e.g., *Sulfolobales* and *Thermoproteales*) possess enzymes for synthesis of G3P, among which the anaerobic glycerol-3-phosphate dehydrogenase subunit A (GlpA) and the aerobic glycerol-3-phosphate dehydrogenase (GlpD) which act as a glycerol-3-phosphate dehydrogenase (G3PDH) (Yokobori et al, 2016). In contrast, glycerol-1-phosphate dehydrogenase (G1PDH) enzymes are present in all archaea (except *Nanoarcheaota*) and are essential for archaeal ether phospholipid synthesis (Yokobori et al, 2016). As far as the distribution of archaeal-like ether lipids in bacteria concerns, in 2008 the a*raM* gene was identified in the bacterium *Bacillus subtilis* which encodes for a G1PDH involved in a phospholipid biosynthesis pathway that yields small amounts of an ether phospholipid with unknown cellular function (Guldan et al, 2008; Guldan et al, 2011; Peterhoff et al, 2014; Lombard and Moreira, 2011). Bacteria belonging to the Fibrobacteres–Chlorobi–Bacteroidetes (FCB) superphylum encode an archaeal pathway for ether-bound isoprenoid membrane lipids in addition to the bacterial fatty acid membrane pathway (Villanueva et al, 2021). Furthermore, an engineered *Escherichia coli* strain, containing archaeal enzymes involved in isoprenoid synthesis and glycerol-phosphate coupling, but lacking an archaeal G1P synthase, resulted in the formation of G1P-based ether lipids and hence a heterochiral membrane, suggesting a yet unidentified G1P synthase in *E. coli* (Caforio et al, 2018). Altogether, these findings further question the widely accepted strict chiral distinction between lipids from archaea and bacteria. Only recently, heterochirality of the glycerol-phosphate backbone has been observed in several prokaryotes. Chiral analysis of phosphatidylglycerol (PG) from *Bacillus amyloliquefaciens*, *B. subtilis*, *Clavibacter michiganensis* and *Geobacillus stearothermophilus* showed both the *R-* and *S-*enantiomers of the glycerol-phosphate backbone to be present in the membrane, in ratios varying from 4:1 to 3:1 for G3P:G1P (Palyzova et al, 2022). Additionally, methyl-phosphatidylethanolamine (PE) and dimethyl-PE from the anaerobic bacteria *Kocuria rhizophila* CCM552, *Raoultella* sp. KDF8, *Pectinatus frisingensis* and *Megasphaera cerevisiae* were determined to be mixtures of both enantiomers (Řezanka et al, 2022). Analysis of *B. cereus*, *B. licheniformis*, and the thermophiles *B. subtilis* subsp. *inaquosorum*, and *B. tequilensis* further identified PG stereoisomers, containing both enantiomers of glycerol-phosphate as polar headgroup as well as the phospholipid backbone (Palyzova et al, 2025).

**Figure 1:**
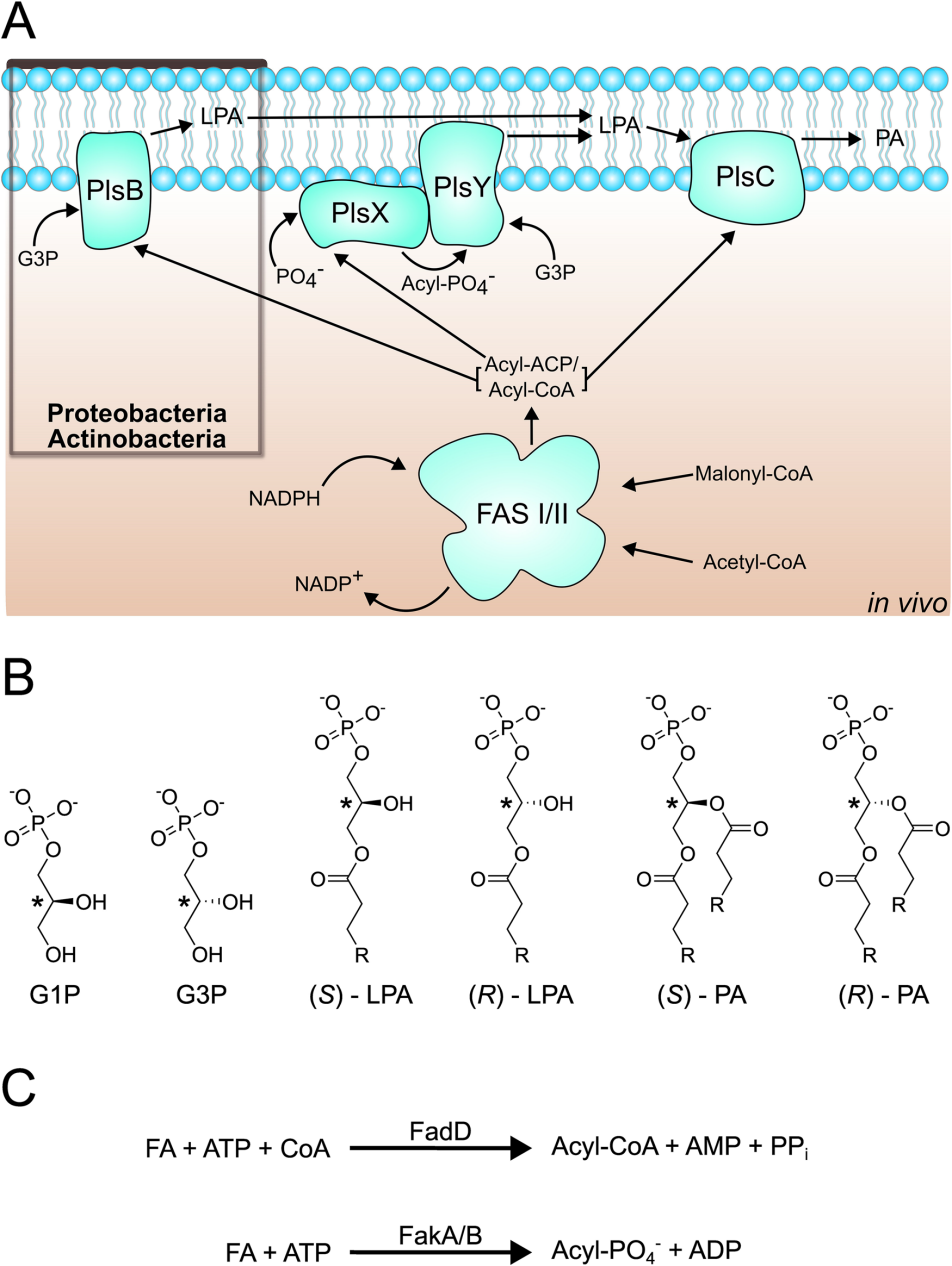
Overview of bacterial phospholipid synthesis. (**A**) Schematic representation of bacterial phospholipid biosynthesis pathway in living cells. The fatty acid synthase (FAS) complex synthesizes the acyl-chain donors acyl-ACP or acyl-CoA, which are then utilized by the acyl-chain transferases PlsX/Y, PlsB and PlsC to form lysophosphatidic acid (LPA) and phosphatidic acid (PA). The PlsX/PlsY system is ubiquitous in the majority of bacterial taxa, while PlsB is largely restricted to proteobacteria and actinobacteria. (**B**) Chemical structures of substrates/products used in this study: *sn*-glycerol-1-phosphate (G1P, (*S*)-configuration), *sn*-glycerol-3-phosphate (G3P, (*R*)-configuration), as well as (*S*)- and (*R*)-isomers of LPA and PA. (**C**) Schematic representation of FadD- and FakA/B-catalyzed reactions for acyl-CoA and acyl-phosphate synthesis, respectively, used in the in vitro experiments of this study. Source data are available online for this figure.

The above stated examples of heterochirality contradict the currently defined classification of lipids among prokaryotes, and question the strictness of the prokaryotic chiral lipid divide (Palyzova et al, 2022; Guldan et al, 2008). Focusing on biosynthesis, the enzymatic mechanisms underlying the formation of lipids with distinct stereoisomeric forms remain unclear. In bacteria, phospholipid synthesis (Fig. 1A) starts with the multi-enzyme protein complex fatty acid synthase (FAS), which utilizes acetyl-coenzyme A (acetyl-CoA) and malonyl-CoA in a cyclic process, thereby enabling stepwise elongation of the fatty acid lipid tail (Fujita et al, 2007). Subsequently, activated fatty acids, *e.g*., acyl-CoA or acyl-acyl carrier protein (acyl-ACP), are attached to glycerol-phosphate that forms the phospholipid backbone. This process starts with the attachment of a first activated fatty acid to the primary carbon of a glycerol-phosphate through an esterification, which is catalyzed by enzymes belonging to the family of glycerol-phosphate acyltransferases (GPAT) (Fig. 1A) (Yoshimura et al, 2007; Zhang and Rock, 2008; Hara et al, 2008). In various bacteria, *e.g*., the Gram-positive *B. subtilis*, the glycerol-3-phosphate acyltransferase PlsY is the only responsible GPAT for this reaction. For this, acyl-ACP, first has to be converted by the phosphate acyltransferase PlsX into acyl-phosphate (acyl-P_i_), which in turn can be utilized by PlsY. Other bacteria, among which many members belonging to the phylum proteobacteria (such as *E. coli*) and actinobacteria, additionally possess the GPAT glycerol-3-phosphate acyltransferase PlsB (Zhang and Rock, 2008). This enzyme can directly utilize acyl-CoA and acyl-ACP. Subsequently, 1-acyl-sn-glycerol-3-phosphate acyltransferase PlsC, belonging to the lysophosphatidic acid acyltransferases (LPAAT) family, catalyzes the attachment of a second activated fatty acid to the *sn*-2 position of the glycerol-phosphate backbone, thereby, converting lysophosphatidic acid (LPA) into phosphatidic acid (PA) (Fig. 1A) (Larson et al, 1980; Heath and Rock, 1998; Yao and Rock, 2013). Bacteria belonging to the phylum Xanthomonadales only contain the PlsB and PlsC enzymes for phospholipid biosynthesis (Zhang and Rock, 2008). All three enzymes are directly involved in the chemical modification of the glycerol-phosphate backbone, hence are potential sources of heterochirality.

In this study, we report on the stereospecificity of the acyltransferases, glycerol-phosphate acyltransferase (GPAT) and lyso-phosphatidic acid acyltransferase (LPAAT) from the Gram-positive *B. subtilis*, as well as the Gram-negative *E. coli*. Through in vitro characterization of purified enzymes, we could show that the GPAT PlsY, as well as the LPAAT PlsC can utilize both stereoforms of the glycerol-phosphate backbone as substrate, whereas the GPAT PlsB is strictly stereoselective towards G3P. These data demonstrate that the GPAT PlsY is the key enzyme responsible for the backbone heterochirality observed in bacterial phospholipids.

## Results

### Acyl-chain–glycerol-phosphate backbone coupling in bacteria

To examine the influence of the glycerol-phosphate stereochemistry on the GPAT PlsY and PlsB and the LPAAT PlsC, we designed an in vitro setup in which the activity of these enzymes can be tested under controlled conditions. For the enzymatic assay, besides glycerol 1-phosphate (G1P) and glycerol 3-phosphate (G3P) (Fig. 1B), the substrates acyl-ACP and acyl-CoA are also required. However, acyl-ACP is not commercially available and structurally unstable in solution, which complicates chemical synthesis. Hence, an alternative approach is required. Naturally, FAS yields the end-product acyl-ACP (Fig. 1A), which is then converted into acyl-P_i_ by PlsX. Acyl-P_i_ is chemically unstable and is immediately utilized as a substrate by PlsY. As an alternative, the fatty acid kinase (Fak) A and B from *B. subtilis* can be used for acyl-P_i_ biosynthesis (Fig. 1C). FakA/B is a coupled enzyme system naturally involved in the activation of free fatty acids for phospholipid synthesis (Parsons et al, 2014; Machinandiarena et al, 2020). In the presence of ATP, FakA/B can directly utilize and activate free fatty acids through phosphorylation, thereby synthesizing the PlsY substrate acyl-P_i_ circumventing the need for PlsX, which was previously shown not to be needed for PlsY activity (Lu et al, 2006). In a similar approach, the long chain fatty acid CoA ligase (FadD) can be used to generate acyl-CoA (Fig. 1C). By utilizing free fatty acids and CoA, FadD catalyzes acyl-CoA formation in an ATP driven reaction, which serves as a substrate for PlsB, as well as PlsC (Exterkate et al, 2018). The *B*. subtilis PlsY and PlsC, as well as the *E. coli* PlsY, PlsB and PlsC were cloned into overexpression plasmids (Table 1), and a Strep-tag® II or a histidine-tag was added to enable purification by using respectively StrepTactin® or Ni+ nitrilotriacetic acid (NTA) resin.

**Table 1:** Cloning and expression vectors used in this study.

Plasmid	Description	Reference
pET-Duet	Expression vector (Amp^R^), T7 promoter, lacI	Addgene
pET_BsFakA_His	pETDuet with *B. subtilis* FakA, also known as yloV, with N-terminal His tag Cloned using primers FakA_For and FakA_Rev and BamHI and EcoRI restriction sites.	This study
pET_Bs_FakB_His	pETDuet with *B. subtilis* FakB, also known as degV, with N-terminal His tag. Cloned using primers FakB_For and FakB_Rev and BamHI and EcoRI restriction sites.	This study
pET_BsFakA_StrepII	*fakA* gene with N-terminal Strep-tag® II from *B. subtilis* cloned into pET-Duet vector using primers Strep_FakA_fwd/rev	This study
pET_BsFakB_StrepII	*fakB* gene with N-terminal Strep-tag® II from *B. subtilis* cloned into pET-Duet vector using primers Strep_FakB_fwd/rev	This study
pSOI	Expression vector (Amp^R^), araBAD promoter, araC	Bakkes et al, 2010
pSOI_EcPlsB_StrepII	*plsB* gene from *E. coli* MG1655 linked via TEV-GSG-linker to a C-terminal Strep-tag® II was cloned from pME002 (Exterkate et al, 2018) into pSOI vector. Backbone for pSOI and plsB gene were amplified using primer pSOI_backbone_fw/rev and plsB_gene_fw/rev. Inserts and backbone were fused via Gibson assembly using fusion primer pSOI_fusion_plsB_10xHisTag fw/rev. Finally, His-Tag was exchanged through Strep-tag® II using primer pSOI_TEV_Strep_fw/rev	This study
pSOI_EcPlsC_StrepII	*plsC* gene from *E. coli* MG1655 linked via GSG-linker to a C-terminal Strep-tag® II was cloned from pME003 (Exterkate et al, 2018) into pSOI vector. Backbone for pSOI and plsC gene were amplified using primer pSOI_backbone_fw/rev and plsC_fw/ plsB_gene_rev. Inserts and backbone were fused via Gibson assembly using fusion primer pSOI_fusion_plsC_10xHisTag_fw/rev. Finally, His-Tag was exchanged through Strep-tag® II using primer pSOI_TEV_Strep_fw/rev	This study
pRSF-Duet	Expression vector (Kan^R^), T7 promoter, lacI	Addgene
pRSF_EcPlsY_StrepII	*plsY* gene from *E. coli* linked via GSGA linker to a N-terminal Strep-tag® II cloned into pRSF vector using primers Strep_EcPlsY_fwd/rev	This study
pRSF_StrepII_FadD	*fadD* gene from *E. coli MG1655* linked via GSGA linker to a N-terminal Strep-tag® II cloned into pRSF vector using primers FadD_Strep_fwd/rev. Previous His-Tag from pME001 template (Exterkate et al, 2018) was removed using primer FadD_His6del_fwd/rev	This study
pRSF_BsPlsY	*plsy* gene from *B. subtilis* to a C-terminal His_6_ – Tag cloned into pRSF vector using primers MHO-134/BsPlsY_For and MHO-135/BsPlsY_Rev	This study
pRSF_BsPlsC	*plsc* gene from *B. subtilis* to a C-terminal His_6_ – Tag cloned into pRSF vector using primers MHO-157/BsPlsC_For and MHO-158/BsPlsC_Rev	This study

### PlsY can utilize both stereo-forms of glycerol-phosphate as substrate

**Figure 2:**
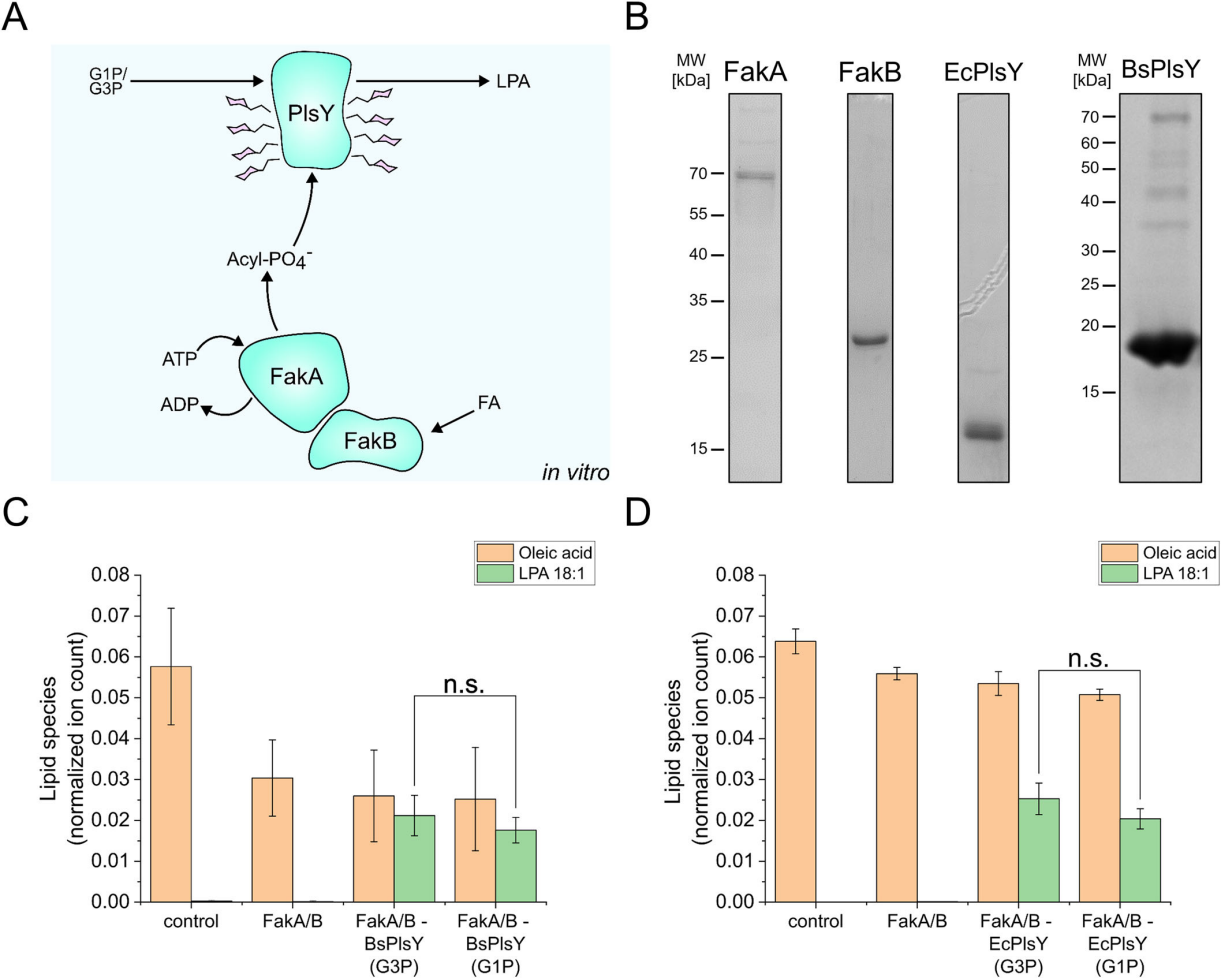
In vitro activity of purified PlsY for lysophosphatidic acid (LPA) synthesis. (**A**) Schematic representation of the FakA/B – PlsY coupled setup in detergent. (**B**) Coomassie-stained SDS-PAGE gel of purified enzymes. In vitro activity of (**C**) *B. subtilis* PlsY (BsPlsY), (**D**) *E. coli* PlsY (EcPlsY) in presence of glycerol-3-phosphate or glycerol-1-phosphate as indicated and compared to control reactions containing G3P and ATP omitting PlsY (FakA/B) or all enzymes (control). Levels of oleic acid and LPA 18:1, detected with LC-MS (Table 3), were corrected for the internal standard n-dodecyl β-D-maltoside (DDM). Data are mean (average) ± SD (*n* ≥ 3, biological replicates). Unpaired *t* test analysis on LPA levels shows no significant difference (n.s.) for BsPlsY (*P* = 0.3491) or EcPlsY (*P* = 0.2141). Source data are available online for this figure.

To examine the stereospecificity of PlsY, a coupled reaction with FakA/B was performed for which purified proteins were employed (Fig. 2A). After affinity chromatography, SDS-PAGE analysis was performed to analyze the purity. For all samples, the majority of eluted protein was formed by the enzyme of interest (Fig. 2B: lane 1 FakA, 62 kDa; lane 2 FakB, 34 kDa), with only minor contaminations. Next, the purified enzymes were used to step-wise build-up the FakA/B – PlsY coupled reaction. Although PlsY is located in the membrane, its activity levels in detergent are comparable (Lu et al, 2007). Hence, we performed the FakA/B – PlsY coupled reaction in detergent, thereby avoiding potential differences in membrane reconstitution efficiency and topology between the *B. subtilis* PlsY (BsPlsY) and *E. coli* PlsY (EcPlsY). First oleic acid was incubated with FakA/B in the presence of G3P and ATP (Fig. 2C). Although, the product acyl-phosphate (Acyl-P_i_) is not stable enough for liquid chromatography- mass spectrometry (LC-MS) detection, decreasing levels of the substrate oleic acid, indicate functional FakA/B. Subsequent addition of BsPlsY to the reaction mix resulted in the production of LPA, thereby verifying the successful coupling. Note that each lipid class ionizes differently, so only relative ion counts within the same class can be compared. Remarkably, the addition of glycerol 3-phosphate, as well as glycerol 1-phosphate, resulted in the substantial production of LPA, with only minor differences in quantities over time. Hence, BsPlsY seems to be unspecific towards the stereochemistry of the glycerol-phosphate backbone. Next, the stereospecificity of EcPlsY was examined (Fig. 2D). Similar to BsPlsY, EcPlsY can utilize both G1P and G3P, resulting in the synthesis of two stereospecific version of LPA. Although for both EcPlsY and BsPlsY the (*R)* enantiomer of LPA seems to be produced in slightly higher yields, it appears there is no major preference of either PlsY enzyme for G3P/G1P as substrate.

### PlsB is stereospecific towards glycerol 3-phosphate

**Figure 3:**
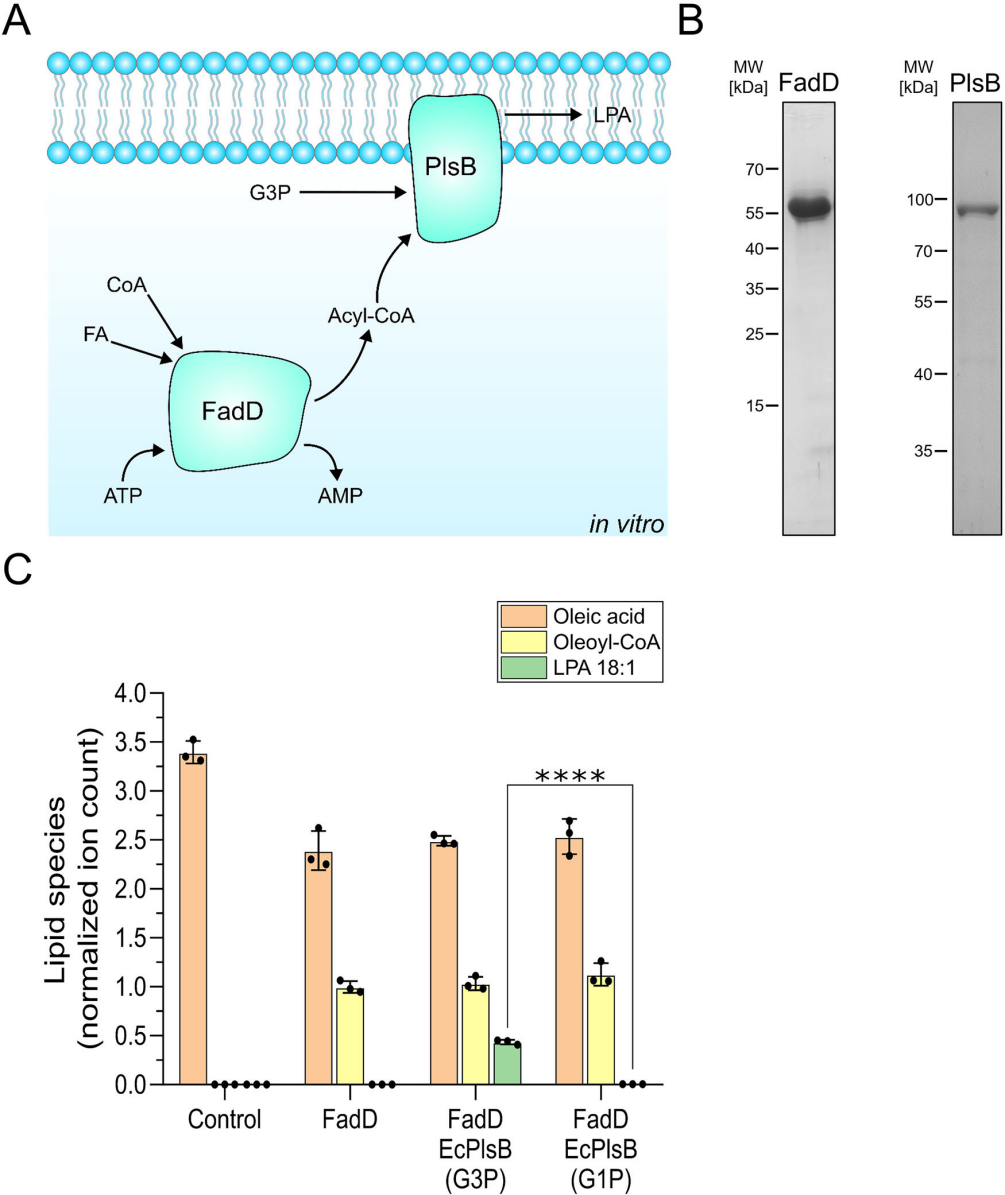
In vitro activity of purified PlsB for lysophosphatidic acid (LPA) synthesis. (**A**) Schematic representation of the FadD – PlsB coupled setup in liposomes. (**B**) Coomassie-stained SDS-PAGE gel of purified enzymes. (**C**) In vitro activity of *E. coli* PlsB (EcPlsB) in presence of glycerol-3-phosphate or glycerol-1-phosphate as indicated, and compared to control reactions containing G3P and ATP omitting PlsB (FadD) or all enzymes (control). Levels of oleic acid, oleoyl-CoA and LPA 18:1, detected with LC-MS (Table 3), were corrected for the internal standard DOPE. Data are mean (average) ± SD (*n* = 3, biological replicates). Unpaired *t* test analysis on LPA levels is significant (****P = <0.0001). Source data are available online for this figure.

Although for some bacteria, like *B. subtilis*, PlsY is the only GPAT, other bacteria, like *E. coli*, possess a second GPAT enzyme, called PlsB, for LPA synthesis. Therefore, we also tested the stereospecificity towards glycerol-phosphate for this enzyme. Physiologically, PlsB uses acyl-ACP or acyl-CoA, which make it suitable for a coupled reaction with the acyl-donor producing enzyme FadD (Fig. 3A) (Exterkate and Driessen, 2019; Exterkate et al, 2018). SDS-PAGE analysis after affinity purification and size exclusion chromatography showed a prominent band upon purifying FadD around 64 kDa. Likewise, high purity was observed for PlsB (94 kDa) (Fig. 3B). As PlsB is not active in detergent, and requires a membrane for its activity, the protein was reconstituted into liposomes (di-oleoyl-phosphatidylglycerol (DOPG): di-oleoyl-phosphatidylethanolamine (DOPE): di-oleoyl-phosphatidylcholine (DOPC), molar ratio 1:1:1), prior to testing its activity. In a similar stepwise approach, first FadD was incubated with oleic acid, CoA, G3P and ATP (Fig. 3C). As expected, oleic acid was converted into the product oleoyl-CoA, thereby confirming FadD activity. In a next step, PlsB proteoliposomes were added, which resulted in the formation of substantial amounts of LPA. In contrast, when G3P was replaced for G1P, only trace amounts of LPA were detected, indicating that PlsB is highly stereospecific towards G3P.

Summarizing, these data show that the GPAT PlsY proteins from both bacterial species are able to utilize both G3P and G1P as lipid backbone substrate, whereas the GPAT PlsB (present in *E. coli*, but not in *B. subtilis*) is stereospecific for G3P. As PlsB is the dominant GPAT in *E. coli*, it is expected that this bacterium contains mostly phospholipids with G3P as lipid backbone (Yoshimura et al, 2007). Indeed, by using chiral-phase high-performance liquid chromatography (HPLC) and electrospray ionization–mass spectrometry (ESI/MS), it was determined that *E. coli* phosphatidylglycerol (PG) only contains the *R* configuration of the lipid backbone (Itabashi and Kuksis, 1997). In contrast, PG from *B. subtilis* is a mixture of both lipid backbone stereoforms (Palyzova et al, 2022). As in this microorganism, PlsY is its sole GPAT, the observed in vivo heterochirality is in line with our in vitro observations. These results potentially have big implications for our current understanding of bacterial phospholipid biosynthesis. Most of the current knowledge is based on the model organism *E. coli*, which like many proteobacteria contain PlsB as well as PlsY. On the other hand, the majority of the bacteria only contain the PlsY (Zhang and Rock, 2008). Hence, a heterochiral membrane might be the predominant form in the bacteria, provided that the heterochirality of PlsY is conserved and appropriate mechanisms for glycerol 1-phosphate biosynthesis are present as well. G1PDH, responsible for the synthesis of G1P, was previously thought to be exclusive to Archaea. However, the bacterial equivalent AraM is found in some bacterial species, notably within the *Bacillus* genus. It should be noted though that it is not ubiquitous across all bacteria. At present, there is no other mechanism of G1P biosynthesis known, but our previous work with an engineered *E. coli* strain that synthesizes archaeal ether phospholipids, demonstrated that other mechanism(s) of G1P biosynthesis must exist in *E. coli* and possibly other bacteria, as archaeal lipids with the correct chiral structure could be produced even in the absence of AraM (Caforio et al, 2018).

### PlsC utilizes both stereo-forms of LPA

**Figure 4:**
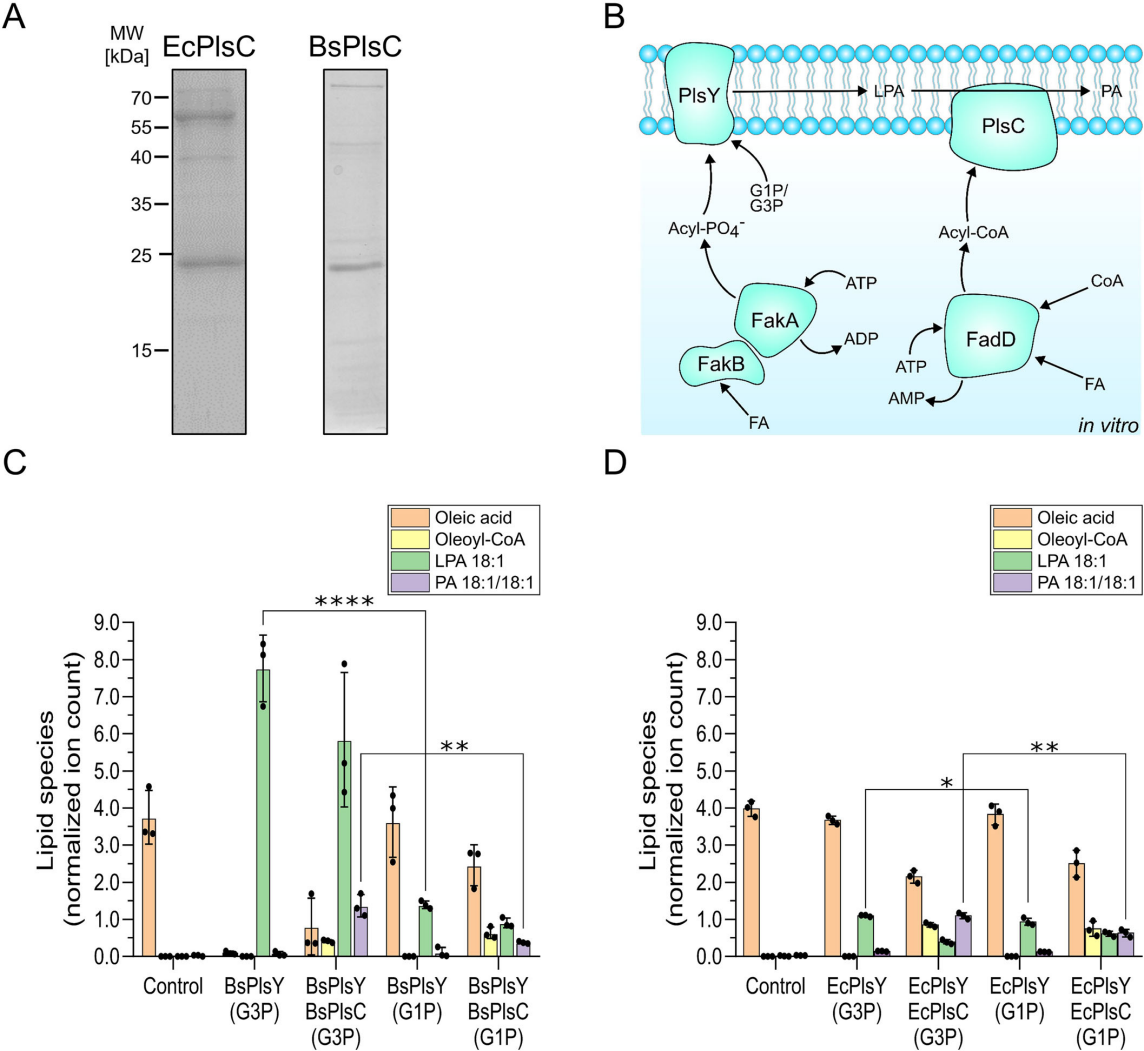
In vitro activity of purified PlsY and PlsC for phosphatidic acid (PA) synthesis. (**A**) Coomassie-stained SDS-PAGE gel of PlsC from *B. subtilis* and *E. coli*. (**B**) Schematic representation of the coupled FakA/B - PlsY setup for LPA synthesis, combined with the subsequent FadD – PlsC mediated conversion into PA in liposomes. (**C**) In vitro activity of *B. subtilis* (BsPlsY), with or without PlsC (BsPlsC), in presence of either glycerol-3-phosphate (G3P) or glycerol-1-phosphate (G1P), compared to a control containing no enzymes and G3P as substrate. Levels of oleic acid, oleoyl-CoA, LPA 18:1 and PA 18:1/18:1, detected with LC-MS (Table 3), were corrected for the internal standard DOPE. Data are mean (average) ± SD (*n* = 3). Unpaired *t* test analysis on LPA levels (****P = <0.0001) and PA levels (**P = 0.0020), shows a significant difference. (**D**) In vitro activity of *E. coli* (EcPlsY), with or without PlsC (EcPlsC), in presence of either glycerol-3-phosphate (G3P) or glycerol-1-phosphate (G1P), compared to a control containing no enzymes and G3P as substrate. Data are mean (average) ± SD (*n* = 3, biological replicates). Unpaired *t* test analysis on LPA levels (*P = 0.0236) and PA levels (**P = 0.0017), shows a significant difference. Source data are available online for this figure.

While LPA is the first membrane integrated intermediate in phospholipid biosynthesis, a second acyl-chain must be attached to synthesize the phospholipid headgroup precursor PA. This reaction is catalyzed by the bacterial lyso-phosphatidic acyltransferase (LPAAT) PlsC, which is universally present throughout the entire bacterial domain of life (Zhang and Rock, 2008). As PlsC’s catalytic activity also directly involves the glycerol-phosphate backbone, its stereospecificity was analyzed as well. Like PlsB, the activity of PlsC is severely impaired in detergent (Exterkate et al, 2018), hence it requires reconstitution into a membrane to achieve optimal function. SDS-PAGE analysis after affinity purification and Sec chromatography of EcPlsC reveals two major protein species slightly below 25 kDa and slightly above 55 kDa (Fig. 4A). Western blot analysis revealed both bands contain PlsC, possibly representing the monomeric and dimeric form of the protein (Appendix Fig. S1A). Noteworthy, the monomer of PlsC runs lower on SDS-PAGE than its theoretical molecular weight (29 kDa), in accordance with the literature (Scott et al, 2016). BsPlsC is detected at its expected position (theoretical molecular weight of BsPlsC is 23 kDa), with minor contaminants (Fig. 4A). The two PlsC enzymes were reconstituted into liposomes containing its direct substrate LPA (DOPG:DOPE:DOPC:LPA, molar ratio 3:3:3:1), and incubated in the presence of FadD, oleic acid, CoA and ATP, required for the synthesis of the other substrate acyl-CoA. As a result, PA was synthesized, which was not observed in control reaction or when PlsC was left out of the reaction (Appendix Fig. S1B–D). Although BsPlsC showed lower activity than EcPlsC, the LC-MS analysis shows that for both enzymes the levels of synthesized PA are clearly distinguishable from the background. Hence, this setup would be suitable to test the stereospecificity of both PlsCs, but LPA containing a G1P backbone confirmation is not commercially available. Therefore, an alternative setup was designed that include the earlier developed FakA/B- PlsY coupled reaction (Fig. 4B). In this reaction scheme, the FakA/B – PlsY reaction is supplemented with either G1P or G3P, resulting in the production of respectively the (*S)* and (*R)* enantiomer of LPA. In a next step, PlsC can then utilize either of these two stereo-forms of LPA, together with oleoyl-CoA provided by FadD. As PlsC requires a membrane for its activity, PlsY was also (co-)reconstituted into liposomes, such that the synthesized LPA will be available for PlsC. Starting with membrane reconstituted BsPlsY, synthesis of LPA was observed in presence of G1P and G3P, as expected (Fig. 4C). However, the levels of (*R*)*-*LPA (containing a G3P lipid backbone) were significantly higher compared to the levels of (*S*)-LPA, a difference that was not observed for the enzyme in detergent (Fig. 2C). Upon addition of FadD and PlsC, MS-analysis further revealed the production of oleoyl-CoA and PA (Fig. 4C). Similarly, the levels of produced PA are significantly higher in the presence of G3P compared to G1P. Although this seems to indicate that BsPlsC prefers (*R*)-LPA as substrate, we cannot rule out this result is partially impacted by the lower availability of (*S*)-LPA. Also, with the membrane reconstituted EcPlsY, both enantiomers of LPA were synthesized when supplied with G3P or G1P (Fig. 4D). Again, the levels of produced (*R*)*-*LPA were significantly higher than (*S*)*-*LPA, but to a much lesser extent as for the Bacillus enzyme. Moreover, addition of FadD and PlsC led to significantly higher levels of (*R*)-PA (G3P) compared to (*S*)-PA (G1P), similar to BsPlsC (Fig. 4D).

Our data shows that both the *E. coli* and *B. subtilis* PlsC can utilize both stereoforms (*R)* and (*S)* of LPA. Interestingly, both PlsC proteins appear to synthesize more (*R)*-configurated PA than the other stereoform, suggesting that LPA containing the G3P backbone is the preferred substrate. Similarly, PlsY also seems to have a higher affinity for G3P. However, to verify this assumed enhanced affinity of PlsC for G3P/(*R)*-LPA, proper kinetic analysis is required. The current in vitro system is, however, unsuitable for such analysis, due to the coupling of enzymes. Nevertheless, this observation is in line with the 3:1 ratio of *R*-configurated lipid backbone over *S*-configurated lipid backbone reported for several Gram-positive bacteria (Palyzova et al, 2022).

### Engineering distinct acyl-chain configurations of phosphatidic acid

**Figure 5:**
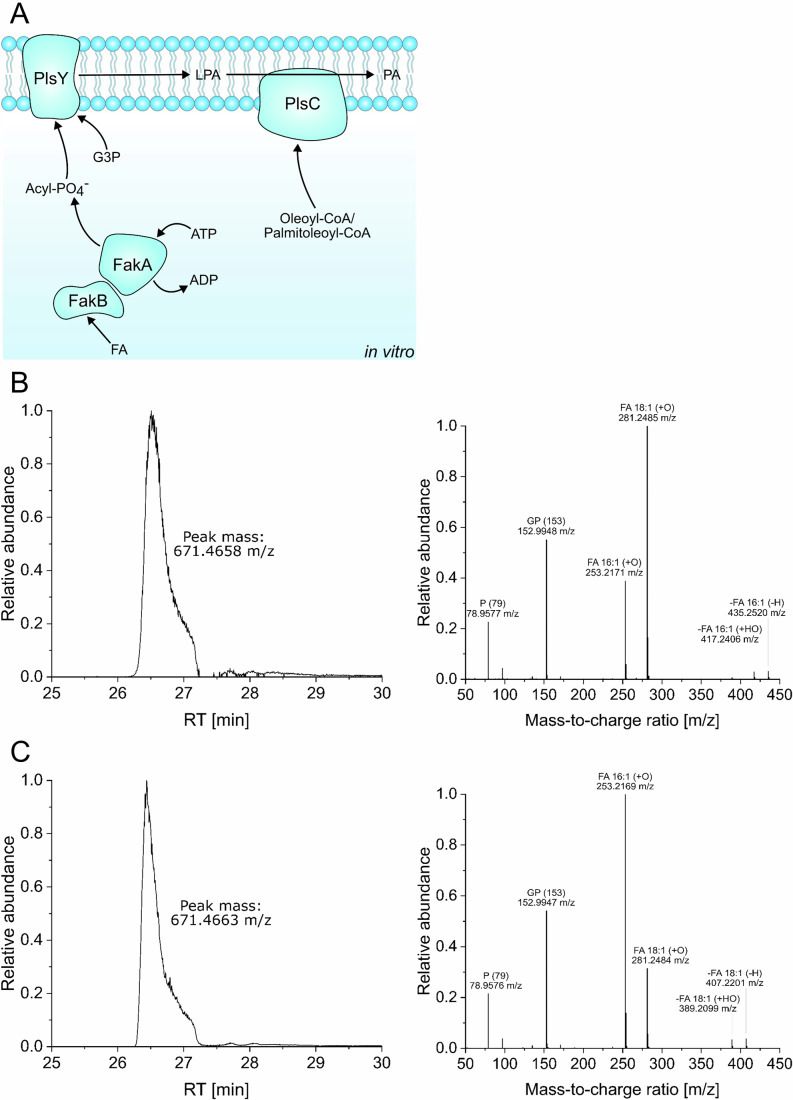
Engineering specifi c phospholipid tail confi guration (**A**) Schematic representation of in vitro PlsY–PlsC coupled reaction to engineer lipid tail specificity. Palmitoleic acid (FA 16:1) was incubated with oleoyl-CoA (18:1), or oleic acid (FA 18:1) incubated with palmitoleoyl-CoA (16:1), both resulting in the synthesis of phosphatidic acid (PA 34:2). (**B**) LC-MS analysis identifying the precursor mass (*m/z* 671.4658 [M-H^−^]) and corresponding MS/MS fragments of PA 18:1/16:1. (**C**) LC-MS analysis identifying the precursor mass (*m/z* 671.4663 [M-H^−^]), and corresponding MS/MS fragments of PA 16:1/18:1. LC-MS identification of the lipid species is specified in Table 3 and Appendix Fig. S3. Source data are available online for this figure.

The activity of GPAT and LPAAT described above concerned solely the specificity for the glycerol-phosphate substrate. However, these enzymes are also directly involved in the incorporation of the lipid tails. The acyl-chain specificity of PlsB, PlsC and PlsY has been extensively described before, both in vitro and in vivo (Rock et al, 1981; Yao and Rock, 2013). Although a wide variety of different acyl-chains can be utilized by all the enzymes, clear preferences for chain length and/or the number of double bonds are observed in vivo. By designing a phospholipid biosynthesis cascade, based on FadD, PlsB and PlsC, we could previously show that a wide variety of different PA species can be synthesized simultaneously when supplied with a fatty acid mixture varying in acyl chain length and degree of unsaturation (Exterkate et al, 2018). However, in this in vitro system actual control over the *sn*-1 and *sn*-2 acyl-chain configuration is lacking. The newly developed phospholipid biosynthesis cascade based on FakA/B–PlsY and PlsC would allow for such control, as FakA/B synthesizes acyl-P_i_ which can only be used by PlsY, whereas FadD synthesizes acyl-CoA which can only be utilized by PlsC (Fig. 5A). In this way, the acyl-chain configuration of PA can be fully controlled. As a proof of principle, we designed a setup in which oleic acid (C18:1) and palmitoleic acid (C16:1) served as lipid tails. To show that in our in vitro setup PlsY, as well as PlsC, can utilize both acyl chains, we performed individual activity assays (Appendix Fig. S2A,B), as well as combined (Appendix Fig. S2C). We decided to use the PlsY and PlsC from *E. coli*, as these enzymes showed overall the highest activity. Although a preference for a C16:1 lipid tail is observed for PlsY as well as PlsC, both enzymes can incorporate oleic acid (C18:1), as well as palmitoleic acid (C16:1) as acyl chains. Next, control over the *sn*-1, *sn*-2 acyl-chain configuration was introduced. For this, a reaction mixture containing FakA/B, PlsY, PlsC and oleic acid (C18:1) was incubated with palmitoleoyl-CoA, which was priorly synthesized by FadD. Alternatively, a reaction mixture containing FakA/B, PlsY, PlsC and palmitoleic acid (C16:1), was incubated with FadD-synthesized oleoyl-CoA. In both scenarios, a PA C34:2 will be synthesized, but with alternating C18:1 or C16:1 on the *sn*-1 and *sn*-2 position. Indeed, MS analysis shows the detection of a molecule with mass 671.466 m/z for both scenarios, corresponding to PA C34:2 [M-H]^−^ (Fig. 5B,C). However, the MS2 data shows two distinct fragmentation patterns. In both cases, fragments from both fatty acid lipid tails (FA 16:1( + O) and FA 18:1( + O)), as well as the glycerol-P_i_ (GP) lipid backbone and phosphate (P) itself were identified, confirming that in both cases the lipid species is a PA with C18:1 and C16:1 as lipid tails. However, in the first reaction mix, two additional fragments, corresponding to LPA 18:1 (−FA 16:1(−H) and -FA 16:1( + HO)) were identified, indicating the C18:1 is located at the *sn*-1 position (PA 18:1/16:1) (Fig. 5B). Vice versa, the second reaction mixture contains the LPA 16:1 fragments (-FA 18:1(−H) and −FA 18:1( + HO)), corresponding to C16:1 at the *sn-*1 position (PA 16:1/18:1) (Fig. 5C). Furthermore, the intensity ratio of both FA fragments also altered, further confirming two positional PA isomers. Noteworthy, we made the rare observation that the *sn-*1 FA fragment is more abundant than the *sn*-2 FA fragment. To confirm the accuracy, we also analyzed the fragmentation spectrum of a phosphatidic acid standard (PA 15:0/18:1(d7)), which showed the exact same pattern (Appendix Fig. S3). Although this system serves only as a proof-of-principle of PA acyl-chain configuration control, its potential to be applied in more advanced bottom-up synthetic lipid engineering approaches is evident. In that respect, multiple in vitro phospholipid biosynthesis cascades have already been developed, yielding a variety of different phospholipid products, but definition of the specific acyl-chain composition is still lacking (Eto et al, 2022; Blanken et al, 2020; Bailoni et al, 2024). Our developed system enables such control, as the GPAT PlsY and LPAAT PlsC utilize distinct acyl-chain donors: acyl-phosphate and acyl-CoA, respectively. In natural cell membranes, the *sn*-1 and *sn*-2 position on the glycerol lipid backbone is often specific and tightly controlled, involving a variety of processes, such as lipid remodeling, lipid signaling, and membrane packing (Angala et al, 2023; Lee et al, 2024). Model membranes containing lipids with a specific, yet adaptable, acyl-chain configuration provide an additional tool for studying these processes in a controlled environment.

## Discussion

The strict chiral distinction between phospholipids from the archaeal and bacterial/eukaryotic domains of life is widely reported and considered textbook knowledge. At the same time, recent scientific developments have led to a detailed analysis of the phospholipid species of an increased number of organisms, that also included the backbone chirality (Caforio et al, 2018; Kropp et al, 2022; Palyzova et al, 2022). Our work fits in with these recent studies suggesting the lipid divide is not quite as cut and dried. Traits thought to be characteristic of archaeal membrane lipids are found in (specific taxonomic groups of) bacteria and vice versa. Some of these traits may have been the result of horizontal gene transfer or convergent evolution that occurred after the lipid divide. Therefore, it remains uncertain if LUCA possessed both the genes for biosynthesis of archaeal and bacterial ether and ester-bonded phospholipids, and whether the extant bacterial-archaeal differences are due to differential loss (Lombard et al, 2012; Lombard and Moreira, 2011). Alternatively, LUCA might have had geochemically synthesized lipids, and the evolutionary invention of distinct bacterial and archaeal phospholipids occurred independently in the stem lineages that gave rise to the Achaea and Bacteria (Martin and Russell, 2003; Sojo et al, 2014; Koga et al, 1998). Irrespective of the exact mechanism of the lipid divide, the PlsY enzyme, that is ubiquitously present in Bacteria (with notable exception of the phylum Xanthomonadales), must have been a rather unselective enzyme for glycerol-phosphate already early in evolution. Further, ether bonded phospholipids have also been identified in bacteria (Damste et al, 2007; Goldfine, 2022), though mostly represent a minor component of the membrane. To date, there is no proof of bacterial G3P-based lipids with isoprenoid chains, even though isoprenoid synthesis is also common in bacteria. Phylogenetic analysis show extensive interdomain horizontal transfer of membrane phospholipid biosynthetic genes, primarily from Archaea to Bacteria but also in the opposite direction (Coleman et al, 2019). Most striking in this regard is that meta-genome analysis of Asgard archaea revealed the presence of many bacterial lipid biosynthetic genes, notably *plsY* and *plsC* but not *plsB* (Coleman et al, 2019; Manoharan et al, 2019). While these archaea do not seem to contain genes that could generate G3P (namely homologs of *glp*, *glpK*, *ugpQ* which function in glycerol metabolism) we have shown G3P presence is not strictly required for PlsY function. Initial lipidomic analysis of the only cultures representative of Asgard Lokiarchaeota presented proof only of archaeal phospholipids (Imachi et al, 2020), but the lipid chemical processing and analysis method used might have overlooked bacterial ester-bonded phospholipids.

## Methods

**Table:** Reagents and tools table

Reagent/resource	Reference or source	Identifier or catalog number
**Experimental models**		
*E. coli* DH5α	Exterkate and Driessen Labs	N.A.
*E. coli* BL21 (DE3)	Exterkate and Driessen Labs	N.A.
*E. coli* XL-Blue	Exterkate and Driessen Labs	N.A.
**Recombinant DNA**		
pET-Duet	Addgene	Cat: 201022
pSOI	Exterkate Lab	Bakkes et al, 2010
pRSF-Duet	Addgene	Cat: 71340
Other expression vectors	This study	Table 1
**Antibodies**		
N.A.		
**Oligonucleotides and other sequence-based reagents**		
PCR primers	Sigma-Aldrich	Table 2
**Chemicals, enzymes and other reagents**		
Lipids (DOPC, DOPG, DOPE, LPA)	Avanti polar lipids	Cat: 850375, 840475, 850725, 857230
Fatty acids (Oleic acid, palmitoleic acid)	Sigma-Aldrich	Cat: O1008, P9417
Restriction enzymes: NcoI, SacI, BamHI, EcoRI	New England Biolabs	Cat: R3193S, R3156S, R3136S, R3101S
KLD Enzyme Mix	New England Biolabs	Cat: M0554S
Gibson Assembly Mix	New England Biolabs	Cat: E2611L
Ampicillin	Sigma	Cat: A9518
Kanamycin	Roth	Cat: T832.3
Inducer: IPTG, Arabinose	Roth	Cat: CN08.4, 5118.4
Strep-Tactin® 4Flow® Resin	Iba	Cat: 2-1250-010
Ni-NTA Resin	Qiagen	Cat: 30230
Potassium phosphate monobasic (KH_2_PO_4_)	Sigma	Cat: 60220-M
di-Potassium hydrogen orthophosphate trihydrate (K_2_HPO_4_ * 3H_2_O)	VWR	Cat: 88363.290
Desthiobiotin	Sigma	Cat: D1411
Trehalose	Sigma	Cat: T0167
Bradford Reagent	Thermo Fisher Scientific	Cat: 23236
n-dodecyl beta-maltoside (DDM)	Glycon	Cat: D97002
GDN	Anatrace	Cat: GDN101 5 gm
Tris	Roth	Cat: 4855.2
Potassium chloride (KCl)	Supelco	Cat: 1.04936
Imidazole	Sigma	Cat: 56749-1KG
Magnesiumchloride hexahydrate (MgCl_2_ * 6H_2_O)	Roth	Cat: 2189.1
Adenosine 5 - triphosphate disodium salt hydrate (ATP)	Sigma	Cat: A26209
*sn* glycerol 3-phosphate (G3P)	Sigma-Aldrich - Merck	Cat: 94124
*sn* glycerol 1-phosphate (G1P)	Scientific Laboratory Supplies Ltd	Cat: 92034-10MG
1,4-Dithiothreit (DTT)	Roth	Cat: 6908.1
Coenzyme A sodium salt hydrate (CoA)	Sigma	Cat: C3144
Butan-1-ol	VWR Chemicals	Cat: 84709.290
Methanol absolute ULC/MS – CC/SFC	Biosolve	Cat: 136841
Ammonium formate	Sigma	Cat: 70221-100G-F
Acetonitril ULC/MS – CC/SFC	Biosolve	Cat: 012041
2-propanol ULC/MS – CC/SFC	Biosolve	Cat: 162641
**Software**		
MS-analysis software	Thermo scientific	Thermo Xcalibur 2.2 SP1.48
Statistical analysis software	Prism Graphpad Prism	Version 8.0.1
**Other**		
Agilent 1290 Infinity HPLC	Agilent Technologies	
Thermo Q Exactive Plus (Electrospray ionization–mass spectrometer)	Thermo Fisher Scientific	

### Bacterial strain and cloning procedures

All vectors used in this study can be found in Table 1 and all primers in Table 2. To create pRSF_BsPlsY and pRSF_BsPlsC expression vectors *plsY* and *plsC* from *B. subtilis* were amplified by PCR using primers BsPlsY_For/ BsPlsY_Rev and BsPlsC_For/ BsPlsC_Rev, respectively. PCR products were cloned into a pRSF_Duet expression vector using restriction sites NcoI and SacI, this included a C-terminal His_6_ – Tag for Immobilized Metal Affinity Chromatography (Imachi et al) purification. To create FakA/B overexpression vectors *fakA* (also known as *yloV*) and *fakB* (also known as *degV*) from *B. subtilis* were amplified by PCR using primers FakA_For and FakA_Rev and FakB_For and FakB Rev and cloned into a pET-Duet vector using BamHI and EcoRI restriction sites. This created vectors pET-FakA_His and pET_FakB_His, where FakA and FakB have an N-terminal 6xHis tag. These vectors were used as a base to create vectors pET_FakA_Strep and pET_FakB_Strep. For Strep-tag® II introduction, primers Strep_FakA_fwd/Strep_FakA rev and Strep_FakB_fwd/ Strep_FakB _rev (Table 2) were used for nucleotide substitution, mediated by their overhangs. PCR products were afterwards treated with KLD enzyme mix (New England Biolabs).

**Table 2:** Oligonucleotide primers used in this study.

Primers	Primer sequence 5´→ 3´	Restriction site
Strep_FakA_fwd	tgaaaaaggttcaggcAGCCAGGATCCGATGTCTAT	n.a.
Strep_FakA_rev	aactgcgggtggctccaGCTGCTGCCCATGGTATA	n.a.
Strep_FakB_fwd	tgaaaaaggttcaggcAGCCAGGATCCGATGAATATTG	n.a.
Strep_FakB_rev	aactgcgggtggctccaGCTGCTGCCCATGGTATA	n.a.
Strep_EcPlsY_fwd	tccgcagtttgaaaaaTAAGAGCTCGGCGCGCCT	n.a.
Strep_EcPlsY_rev	tggctccagcctgaaccCTCGGGATCCTTTTCGCGC	n.a.
FadD_His6del_fwd	TGAGAGCTCGGCGCGCCT	n.a
FadD_His6del_rev	GGCTTTATTGTCCACTTTGCCGCG	n.a
FadD_Strep_fwd	tgaaaaaggttcaGGCGCCAAGAAGGTTTGGCTT	n.a.
FadD_Strep_rev	aactgcggatggctccaCATGGTATATCTCCTTATTAAAGTTAAAC	n.a.
FakA_For	GAAGGATCCGATGTCTATCAGAACATTAGACG	BamHI
FakA_Rev	GAAGAATTCCTATTCTGCTGAAACTATATACG	EcoRI
FakB_For	GAAGGATCCGATGAATATTGCAGTCGTAACAG	BamHI
FakB_Rev	GAAGAATTCTTATTTAAAACACCAGCAAATTCC	EcoRI
BsPlsY_For	GTTTTCCATGGGCTTAATTGCTTTATTGATTATTTTGG	NcoI
BsPlsY_Rev	GTTTTGAGCTCTTAGTGGTGATGGTGATGATGTAACCATTTTACTTTAGGTTCTG	SacI
BsPlsC_For	GTTTTCCATGGATGTATAAGTTTTGTGCAAATGC	NcoI
BsPlsC_Rev	GTTTTGAGCTCTTAGTGGTGATGGTGATGATGTAGCTGATCAAGTTTATTCTC	SacI
pSOI_backbone_fw	TAAGCTTGGCTGTTTTGG	n.a.
pSOI_backbone_rev	GGTTAATTCCTCCTGTTAGC	n.a.
plsB_gene_fw	ATGGCCGGCTGG	n.a.
plsB_gene_rev	GTGGTGGTGGTGGTGGTGGTGGTGGTGG	n.a.
pSOI_fusion_plsB_10xHisTag fw	ACCACCACCACCACTAAGCTTGGCTGTTTTG	n.a.
pSOI_fusion_plsB_10xHisTag rev	CCAGCCGGCCATGGTTAATTCCTCCTGTTAG	n.a.
pSOI_TEV_Strep_fw	ctggagccatccgcagtttgaaaaaTAAGCTTGGCTGTTTTGGCG	n.a
pSOI_TEV_Strep_rev	cctgaaccctgaaaataaagattctcCTCGAGCCCTTCGCCCTG	n.a
plsC_fw	ATGGTATATATCTTTCGTCTTATTAT	n.a
pSOI_fusion_plsC_10xHisTag_fw	ACCACCACCACCACTAAGCTTGGCTGTTTTG	n.a
pSOI_fusion_plsC_10xHisTag_rev	AATAATAAGACGAAAGATATATACCATGGTTAATTCCTCCTGTTAG	n.a

Small letters indicate primer overhangs for Tag insertion, therefore restriction site not applicable (n.a.). Introduced restriction sites are underlined.

Genes for PlsB, PlsC, PlsY and FadD from *E. coli* were cloned into expression vectors (Table 1) using PCR. For Strep-tag® II insertion primers containing overhangs were used (Table 2). Plasmid ligation was performed using KLD enzyme mix (New England Biolabs). *E. coli* DH5α and XL-1 Blue (Invitrogen) were used as hosts for plasmids. *E. coli* BL21 (DE3) was used for protein overexpression under aerobic conditions at 37 °C and 180 rpm. Antibiotics were used in the following concentrations: kanamycin (30 µg/ml) and ampicillin (100 µg/ml).

### Enzyme expression and purification

Proteins of phospholipid biosynthesis machinery were overexpressed in *E. coli* BL21 (DE3) strain. After reaching an optical density (OD_600_) between 0.6 and 0.8, cells were induced, either with 1 mM IPTG or 1 mM arabinose in case of PlsB and PlsC. After 2 h of expression, cells were separated from supernatant by centrifugation (Avanti JXN-26 Beckman Coulter; Avanti JLA 8.1000 rotor Beckman Coulter (fixed angle), 4000×*g*, 10 min, 4 °C).

Purification of FakA, FakB and FadD (soluble proteins) started with cell disruption (Constant Systems Ltd) including a centrifugation (Avanti J-26 XP Beckman Coulter; JA-25.50 rotor Beckman Coulter (fixed angle), 30,000×*g*, 30 min, 4 °C) step afterwards to separate soluble protein fraction (supernatant) from cell debris. For protein binding, 400 µl of Strep-Tactin® 4Flow® (iba) resin were added to the supernatant of a 2 L expression culture and incubated for 60 min at 4 °C. Afterwards, beads were used for affinity-chromatography within gravity-flow columns. Therefore, beads were washed with total volume of 5 mL washing buffer (50 mM H_2_KPO_4_/HK_2_PO_4_, pH 8) and eluted with 5 mL of elution buffer (50 mM H_2_KPO_4_/HK_2_PO_4_, 5 mM Desthiobiotin, pH 8). Elution fractions were concentrated (Heraeus Megafuge 16 R Thermo Scientific (swing-out), 3000×*g*, 4 °C) till a volume of 500 µl was reached) and stored at −80 °C together with 400 mM Trehalose. Bradford Assay was used for determination of protein concentration.

Purification of EcPlsB, EcPlsC and EcPlsY (membrane proteins) included an initial membrane isolation before purification. Therefore, cells were disrupted and centrifuged to separate supernatant and cell debris. Afterwards, supernatant was centrifuged using ultra-centrifugation (Sorvall® Discovery 90SE Thermo Scientific; Type 70 Ti rotor Beckman Coulter (fixed angle), 180,000×*g* for 60 min and 4 °C) to separate cell membranes from soluble components. Cell membranes (pellet) were resuspended in phosphate buffer (50 mM H_2_KPO_4_/HK_2_PO_4_, pH 8). For solubilization membranes were diluted to 40 mg/ml and 1% of n-dodecyl-ß-D-maltoside (DDM) was added and incubated for 60 min at 4 °C. Solubilized proteins were separated from membrane via a second ultra-centrifugation step. For protein binding 400 µl Strep-Tactin® 4Flow® (iba) were added and purification followed the previous protocol. For washing 50 mM H_2_KPO_4_/HK_2_PO_4_, 0.1% DDM, pH 8 buffer was used, while for elution 50 mM H_2_KPO_4_/HK_2_PO_4_, 0.1%, 5 mM Desthiobiotin, pH 8 was used. In case of PlsB purification 0.05% GDN instead of DDM was used. Elution fractions were concentrated (Heraeus Megafuge 16 R Thermoscientific (swing-out), 3000×*g*, 4 °C) till a volume of 500 µl was reached and stored at −80 °C together with 400 mM Trehalose. Bradford Assay was used for determination of protein concentration.

For BsPlsC and BsPlsY (membrane proteins) purification cell membrane was first isolated. Therefore, cells were disrupted and centrifuged (Avanti J-26 XP Beckman Coulter; JA-25.50 rotor Beckman Coulter (fixed angle), 30,000×*g*, 30 min and 4 °C), to separate supernatant and cell debris. Afterwards, supernatant was centrifuged using ultra-centrifugation (Sorvall® Discovery 90SE Thermo Scientific; Type 70 Ti rotor Beckman Coulter (fixed angle) 180,000×*g* and 4 °C) to separate cell membranes from soluble components. Cell membranes (pellet) were resuspended in Tris buffer (50 mM Tris-HCl pH 8, 100 mM KCl. For solubilization membranes were diluted to 40 mg/ml and 2% of DDM was added and incubated for 60 min at 4 °C. Solubilized proteins were separated from membrane using ultra-centrifugation. For protein binding 400 µl Ni–NTA resin were added and purification followed the previous protocol. For washing Tris Buffer (50 mM Tris-HCl, 100 mM KCl 0.05% DDM, 20 mM imidazole, pH 8) was used, for elution the same buffer was used but with 300 mM imidazole. Elution fractions were concentrated (Heraeus Megafuge 16 R Thermoscientific (swing-out), 3000×*g*, 4 °C) till a volume of 500 µl was reached and stored at −80 °C. Bradford Assay was used for determination of protein concentration.

### Liposome preparation

Chloroform Stocks of the lipids DOPC, DOPE, DOPG and LPA were purchased from Avanti (Avanti Polar Lipids) and pipetted together in absence (DOPC:DOPE:DOPG—1:1:1 molar ratio) or presence of LPA ((DOPC:DOPE:DOPG:LPA—3:3:3:1 molar ratio). Using a rotary evaporator, lipids were dried for 10 min at 400 mbar, 10 min at 300 mbar, 10 min 200 mbar and 10 min at 0 mbar. Dried lipid film was resuspended in 50 mM H_2_KPO_4_/HK_2_PO_4_, pH 8. For formation of liposomes a sonication cycle of 15 s on, 50 s off was repeated for 10–15 times.

### In vitro enzyme activity assays

All in vitro reactions were performed in a total volume of 100 µl. Assays for LPA synthesis using purified BsPlsY were performed using 50 mM Tris-HCl pH 7.5, 20 mM MgCl_2_, 150 mM KCl, 0.1 mM DDM, 10 mM ATP and 0.5 mM oleic acid. Enzyme concentrations of 0.5 µM FakA, 1.0 µM FakB and 1.0 µM BsPlsY were used. Reactions containing PlsY were started with either 10 mM G3P (Sigma-Aldrich—Merck) or G1P (Scientific laboratory supplies Ltd), whereas FakA/B only reactions were started with ATP, and incubated for 30 min at 37 °C while shaking at 650 rpm. LPA synthesis using purified EcPlsY were performed using 50 mM H_2_KPO_4_/HK_2_PO_4_ pH 7, 10 mM MgCl_2_, 2 mM DTT, 0.1 mM DDM, 10 mM ATP and 0.6 mM oleic acid. Enzyme concentrations of 0.5 µM FakA, 1.0 µM FakB and 1.0 µM EcPlsY were used. Reactions were started with either 10 mM G3P or G1P and incubated for 30 min at 37 °C while shaking at 650 rpm.

In vitro reactions focusing on stereospecificity of EcPlsB were performed in 50 mM H_2_KPO_4_/HK_2_PO_4_ pH 7, 10 mM MgCl_2_, 2 mM DTT, 1 mg/ml liposomes (DOPE:DOPC:DOPG 1:1:1), 10 mM ATP, 0.4 mM CoA and 0.6 mM oleic acid. Enzyme concentrations of 0.1 µM EcPlsB were used. Reactions containing PlsB were started with either 10 mM G3P or G1P, whereas FadD only reactions were started with ATP, and incubated for 30 min at 37 °C while shaking at 650 rpm.

Combined in vitro assay containing FakA/B – BsPlsY/ EcPlsY and FadD – BsPlsC/EcPlsY were performed in 50 mM H_2_KPO_4_/HK_2_PO_4_ pH 7, 10 mM MgCl_2_, 2 mM DTT, 1 mg/ml liposomes (DOPE:DOPC:DOPG 1:1:1), 10 mM ATP, 0.4 mM CoA and 0.6 mM oleic acid. Reactions were started by adding 10 mM G3P or G1P. Here, 0.5 µM FakA, 1 µM FakB, 1 µM EcPlsY/ BcPlsY, 1 µM FadD and 1 µM EcPlsC/BsPlsC were used as enzyme concentrations.

Same conditions were used for EcPlsY – EcPlsC mixed fatty acid assay, with additional heat inactivation of FadD reaction at 95 °C for 5 min and subsequent cooling phase for 5 min on ice. As substrates either 0.6 mM oleic acid or 0.6 mM palmitoleic acid were used for single fatty acid reactions, while for mixed fatty acid reaction, a ratio of 1:1 oleic acid: palmitoleic acid was used. Reaction was incubated for 30 min at 37 °C while shaking at 650 rpm.

Lipids were extracted two times using 300 µl *n-*butanol. The organic phase was dried at 55 °C under a stream of nitrogen. Dried lipid film was resuspended in 50 µl methanol and ready for LC-MS analysis.

### FadD–PlsC in vitro assays for PA synthesis

All in vitro reactions were performed in a total volume of 100 µl. For PA 18:1/18:1 synthesis purified FadD and EcPlsC were used under the following conditions: 50 mM H_2_KPO_4_/HK_2_PO_4_ pH 7, 10 mM MgCl_2_, 2 mM DTT, 1 mg/ml liposomes (DOPE:DOPC:DOPG:LPA 3:3:3:1), 0.4 mM CoA and 0.6 mM oleic acid. By adding 10 mM ATP, reaction was started and incubated for 30 min at 37 °C while shaking at 650 rpm.

### Combined reaction of EcPlsY–EcPlsC reaction with mixed acyl chain fatty acids

All in vitro reactions were performed in a total volume of 100 µl. For synthesis of different PA species purified FakA/B – EcPlsY for LPA synthesis were combined with FadD and EcPlsC reaction. Reaction conditions: 50 mM H_2_KPO_4_/HK_2_PO_4_ pH 7, 10 mM MgCl_2_, 2 mM DTT, 1 mg/ml liposomes (DOPE:DOPC:DOPG 1:1:1), 10 mM ATP, 0.4 mM CoA. Reactions were started by adding 10 mM G3P. As substrates either 0.6 mM oleic acid or 0.6 mM palmitoleic acid were used for single fatty acid reactions, while for mixed fatty acid reaction, a ratio of 1:1 oleic acid: palmitoleic acid was used. Reaction was incubated for 30 min at 37 °C while shaking at 650 rpm.

### LC-MS analysis

Samples were analyzed using an Agilent Technologies 1290 Infinity high-performance liquid chromatography (HPLC) system, coupled to a heated electrospray ionization–mass spectrometer (Thermo Q Exactive Plus; Thermo Fisher Scientific). In total, 5 µl was injected into an ACQUITY™ UPLC CSH C18 1.7 µm Column, 2.1 × 150 mm (Waters Chromatography Ireland Ltd) operating at 55 °C with a flow rate of 300 µl/min. Separation of the compounds was achieved by a changing gradient of eluent A (5 mM ammonium formate in water/acetonitrile 40:60, v/v) and eluent B (5 mM ammonium formate in acetonitrile/2-propanol, 10:90, v/v). The following linear gradient was applied: (1) 5% eluent B for 2.5 min, (2) a gradient from 5% to 90% eluent B over 36.5 min, (3) holding for 3 min, (4) returning to 5% eluent B in 0.5 min, (5) and holding for 8 min. The column effluent was injected directly into the Thermo Q Exactive Plus operating in negative ion mode: Spray voltage: 3.20 |kV | , Capillary temperature: 230 °C, S-lens RF level: 50.0, Sheath gas flow: 30, Auxiliary gas flow: 20, Sweep gas flow: 3, Aux gas heater temperature: 380 °C. For MS/MS the DDA method TopN (5) was used. Spectral data constituting total ion counts were analyzed using the Thermo Scientific XCalibur processing software by applying the Genesis algorithm-based automated peak area detection and integration. The total ion counts of the extracted lipid products (Table 3) were corrected for the internal standard DDM (*m/z* 509.3 [M-H]^−^), or DOPE (m/z 742.54 [M-H]^−^) (see respective figure legend), and plotted on the *y* axis as normalized ion count in a bar graph. Due to differences in ionization of different lipid species, only ion counts of a single lipid species can be compared, not between lipid species. To further improve visualization, lipid species were scaled (see respective source files).

**Table 3:** Detected lipid species with LC-MS.

Lipid species	*m/z* [M-H]^−^	Retention time (min)	Diagnostic fragments *m/z* [M-H]^−^
Oleic acid (OA, FA 18:1)	281.25	17.20	-
Palmitoleic acid (FA 16:1)	253.22	12.09	-
Oleoyl-CoA	1030.35	2.56	Oleoyl moiety^a^ (-H2O) – 683.29
Palmitoleoyl-CoA	1002.32	1.84	palmitoleoyl moiety^a^ (-H2O) – 655.26
Oleoyl lysophosphatidic acid (LPA 18:1, LPA)	435.25	4.85	FA 18:1( + O) – 281.25 -FA 18:1 (-H) – 171.01 GP (153) – 152.99 P (79) – 78.96
Palmitoleoyl lysophosphatidic acid (LPA 16:1)	407.22	2.82	FA 16:1( + O) – 253.22 -FA 16:1 (-H) – 171.01 GP (153) – 152.99 P (79) – 78.96
Di-oleoyl phosphatidic acid (DOPA, PA, PA 18:1/18:1)	699.50	28.58	FA 18:1( + O) – 281.25 -FA 18:1( + HO) – 417.24 -FA 18:1(-H) – 435.25 GP (153) – 152.99 P (79) – 78.96
Palmitoleoyl_oleoyl phosphatidic acid (PA 34:2, PA 18:1_16:1, PA 18:1/16:1, PA 16:1/18:1)	671.47	26.45	FA 16:1( + O) – 253.22 FA 18:1( + O) – 281.25 -FA 16:1( + HO) – 417.24 -FA 16:1(-H) – 435.25 -FA 18:1( + HO) – 389.21 -FA 18:1(-H) – 407.22 GP (153) – 152.99 P (79) – 78.96
Di-palmitoleoyl phosphatidic acid (PA 16:1/16:1)	643.43	24.45	FA 16:1( + O) – 253.22 -FA 16:1( + HO) – 389.21 -FA 16:1 (-H) – 407.22 GP (153) – 152.99 P (79) – 78.96
Di-oleoyl phosphatidylglycerol (DOPG, PG)	773.53	27.39	-FA 18:1( + HO) – 491.28 -FA 18:1(-H) – 509.29 FA 18:1( + O) – 281.25 GP(153) – 152.99 HG(PG,171) – 171.01 HG(PG,227) – 227.03
Di-oleoyl phosphatidylethanolamine (DOPE, PE)	742.54	31.05	-FA 18:1( + HO) – 460.28 -FA 18:1(-H) – 478.29 FA 18:1( + O) – 281.25 HG(PE,196) – 196.04
Di-oleoyl phosphatidylcholine (DOPC, PC)	830.59^b^	30.47	-FA 18:1( + HO)-(CH3 + HCOO) – 488.31 -FA 18:1(-H)-(CH3 + HCOO) – 506.32 FA 18:1( + O) – 281.25

*FA* fatty acid, *GP* glycerol-phosphate, *P* phosphate, *HG* Headgroup.

^a^The acyl group + the β-mercaptoethanolamine + the di-phosphopantothenic acid fragment (without the adenosine-monophosphate).

^b^formate adduct *m/z* [M + HCOO]−.

## Supplementary information

Appendix

Peer Review File

Source data Fig. 1

Source data Fig. 2

Source data Fig. 3

Source data Fig. 4

Source data Fig. 5

## Data Availability

The source data of this paper includes SDS-PAGE gels of purified proteins, mass spectrometry raw ion counts of the integrated peak areas of lipid species, and mass spectrometry fragmentation spectra, which are collected in the following Biostudies database record 10.6019/S-BSST3002. Furthermore, Mass-Spectrometry raw files are published as FAIR data under 10.60534/e7zrt-dgx12. The source data of this paper are collected in the following database record: biostudies:S-SCDT-10_1038-S44319-026-00827-z.
