# Supplementary material for: Bacterial lipid synthesizing enzymes PlsY and PlsC utilize both stereo-forms of glycerol-phosphate
Source: EMBO Rep. 2026 Jun 12;27(14):3928–43. doi: 10.1038/s44319-026-00827-z (PMC13400652; doi:10.1038/s44319-026-00827-z)
Supplement: Supplementary file 1 — Appendix [file 44319_2026_827_MOESM1_ESM.pdf]

Appendix

Bacterial lipid synthesizing enzymes PlsY and PlsC utilize both stereo-forms of glycerol-phosphate

Philipp Rieche[1¶], Mirthe Hoekzema[2¶], Sergiy Gan[1], Greetje A. Berrelkamp-Lahpor[2], Adanna W. Ezissi[1], Marten Exterkate[1#] and Arnold J. M. Driessen[2#]

[1] Membrane Biogenesis and Lipidomics, Institute of Biochemistry, Heinrich Heine University, Universitätsstraße 1, 40225, Düsseldorf, Germany
[2] Department of Molecular Microbiology, Groningen Biomolecular Sciences and Biotechnology Institute, University of Groningen, Nijenborgh 7, 9747 AG, the Netherlands

[#] Corresponding authors: marten.exterkate@hhu.de, a.j.m.driessen@rug.nl
[¶] Equal contribution

Table of contents:

The appendix consists of three supplementary figures accompanied with text and materials and methods.

Appendix Figure S1 (page 2)
S1A: Western blot analysis of purified *E. coli* PlsC
S1B: Schematic representation of *in vitro* PA synthesis.
S1C: *In vitro* synthesis of PA catalyzed by PlsC from *B. subtilis.*
S1D: *In vitro* activity of PA catalyzed by PlsC from *E. coli.*

Appendix Figure S2 (page 4)
S2A*: In vitro* synthesis of LPA catalyzed by EcPlsY using fatty acids with varying lengths.
S2B: *In vitro* synthesis of PA catalyzed by EcPlsC using fatty acids with varying lengths.
S2C: *In vitro* synthesis of PA using EcPlsY and EcPlsC with fatty acids of varying chain lengths in a combined assay.

Appendix Figure S3: fragmentation spectrum of the lipid standard PA 15:1/18:1(d7) (page 5)

Engineering the FadD-PlsC coupled system for phosphatidic acid synthesis

To assess the activity of PlsC, a coupled system was designed (Appendix Fig. S1B) with the enzyme FadD, which is involved in the β-oxidation of fatty acids. In a first step, FadD utilizes readily supplied oleic acid, producing oleoyl-CoA. Together with lysophosphatidic acid (LPA 18:1), Oleoyl-CoA then serves as a substrate for PlsC, resulting in the final product phosphatidic acid (PA 18:1/18:1). Indeed, in the presence of CoA and ATP, levels of oleic acid decrease, whereas levels of Oleoyl-CoA arise, indicating FadD activity (Appendix Fig. S1C-D). Next, liposome reconstituted PlsC consisting of DOPG, DOPE, DOPC and LPA 18:1 in a molar ratio of 3:3:3:1, was added to the reaction mix. Although, EcPlsC appears to be more active in our in vitro setup, the presence of either EcPlsC or BsPlsC resulted in significantly elevated PA levels, indicating both enzymes are active (Appendix Fig. S1C-D).

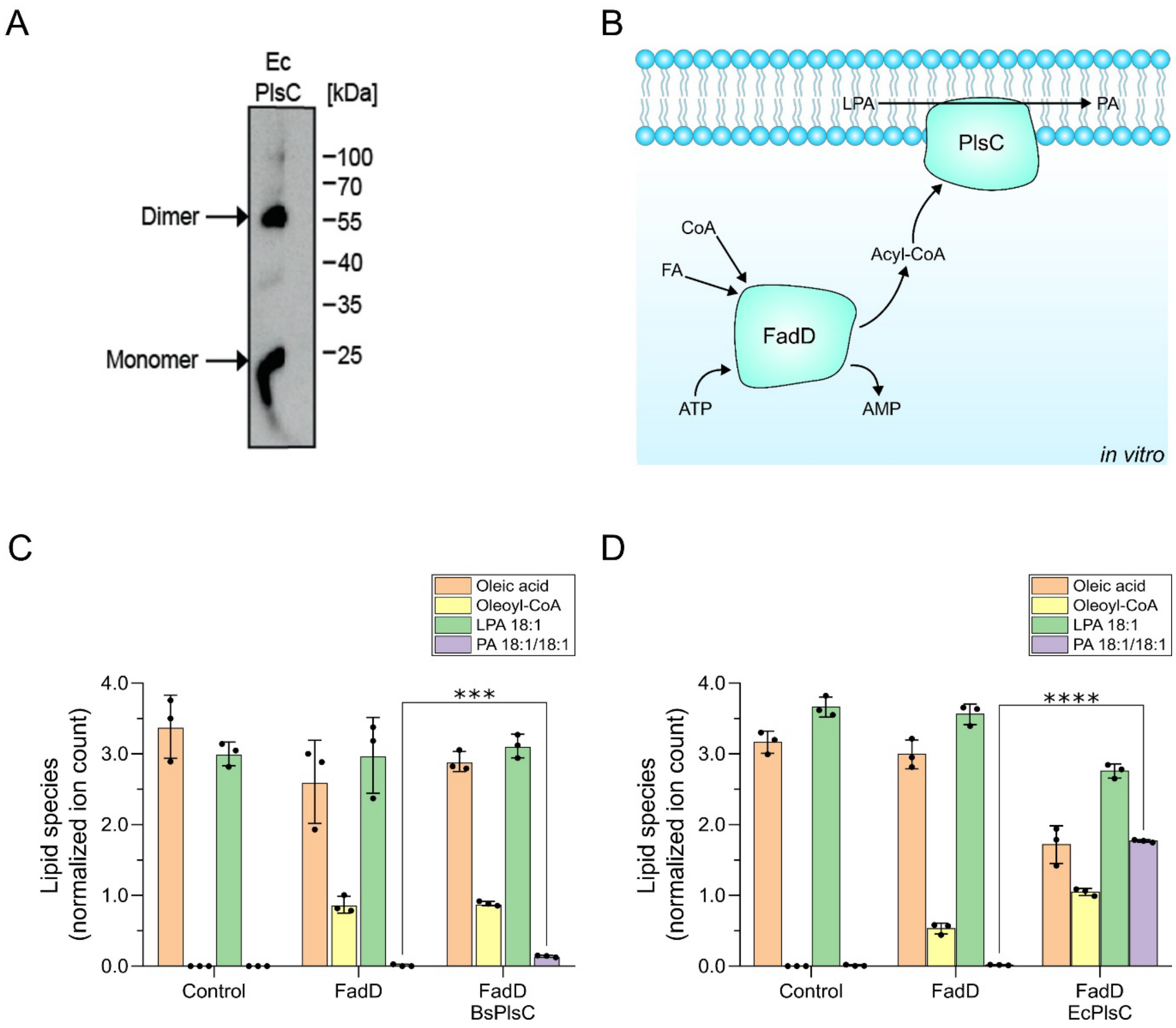

Appendix Figure S1 (A) Western blot analysis of *E. coli* PlsC (EcPlsC) by anti-strep antibody. (B) Schematic representation of the FadD – PlsC combined setup in liposomes. (C) *In vitro* activity of *B. subtilis* PlsC (BsPlsC), compared to control reactions without PlsC (FadD) or no enzymes (control). Levels of oleic acid, oleoyl-CoA, LPA 18:1 and PA 18:1/18:1, detected with LC-MS (table 3), were corrected for the internal standard DOPE. Data are mean (average) ± SD (n = 3). Unpaired t-test analysis on PA levels shows a significant difference for BsPlsC (***p = 0.0005) (D) *In vitro* activity of *E. coli* PlsC (EcPlsC), compared to control reactions without PlsC (FadD) or no enzymes (control). Levels of oleic acid, oleoyl-CoA, LPA 18:1 and PA 18:1/18:1, detected with LC-MS (table 3), were corrected for the internal standard DOPE. Data are mean (average) ± SD (n = 3). Unpaired t-test analysis on PA levels shows a significant difference for EcPlsC (****p < 0.0001).

Acyl chain specificity of membrane reconstituted EcPlsY and EcPlsC

To test the acyl-chain specificity of EcPlsY, the FakA/B-PlsY system was incubated with oleic acid (18:1) and/or palmitoleic acid (16:1). In the presence of ATP and G3P, both fatty acids could be converted into their respective lysophosphatidic acids (Appendix Fig. S2A). Furthermore, adding both fatty acids resulted in the simultaneous production of LPA 18:1 and LPA 16:1. Comparing LPA synthesis with the individual fatty acid feeds, levels of LPA 18:1 are significantly lower in presence of both fatty acids, whereas no significant difference in LPA 16:1 has been observed which implies that the latter LPA species is preferred.

Likewise, the acyl-chain specificity of EcPlsC was examined by using the FadD-PlsC coupled system (Appendix Fig. S1A). As expected, feeding with either oleic acid (18:1) or palmitoleic acid (16:1), resulted in the synthesis of their respective acyl-CoA and PA species (Appendix Fig. S2B). Furthermore, adding both fatty acids resulted in the simultaneous production of PA 18:1/18:1 and PA 18:1/16:1. Comparing PA synthesis with the individual fatty acid feeds, levels of PA 18:1/18:1 are significantly lower in presence of both fatty acids, whereas no significant difference in PA 18:1/16:1 has been observed. Thus, like EcplsY, EcPlsC also prefers the 16:1 acyl-chain as substrate. Indeed, when combining EcPlsY and EcPlsC into one assay, the preference for the palmitoleic acid becomes even more apparent (Appendix Fig. S2C). From the three synthesized version of PA, PA 16:1/16:1 is dominant, whereas moderate levels of PA 18:1_16:1 (the *sn*-position is unknown, which is either PA 18:1/16:1 or PA 16:1/18:1. Hence, PA 18:1_16:1) are produced. Although not clearly visible, trace amounts of PA 18:1/18:1 could be observed as well.

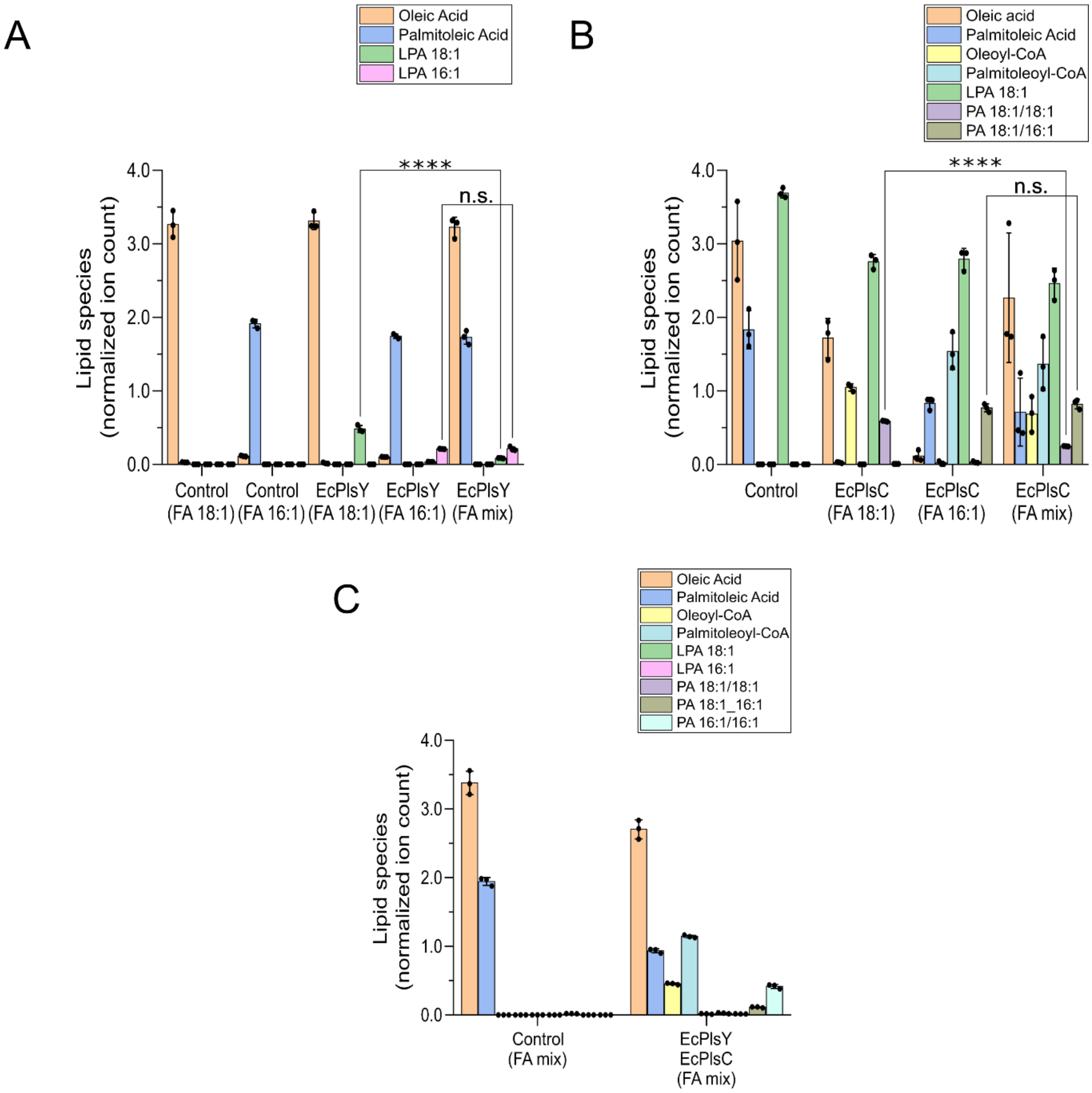

Appendix Figure S2 *In vitro* assays with *E. coli* PlsY and/or PlsC with a variety of fatty acid substrates. (A) *In vitro* activity of *E. coli* PlsY (EcPlsY) in presence of oleic acid (FA 18:1), palmitoleic acid (FA 16:1) or both (FA mix). As a control no enzyme reactions were performed. Synthesized lipid species were analyzed using LC-MS. Levels of species were corrected for the internal standard DOPE. Data are mean (average) ± SD (n = 3). Unpaired t-test analysis shows a significant difference for the levels of LPA 18:1 (****p < 0.0001), but not significant (n.s.) for LPA 16:1 (p = 0.7595) (B) *In vitro* activity of *E. coli* PlsC (EcPlsC) in presence of oleic acid (FA 18:1), palmitoleic acid (FA 16:1) or both (FA mix). As a control no enzyme reactions were performed. Synthesized lipid species were analyzed using LC-MS. Levels of species were corrected for the internal standard DOPE. Data are mean (average) ± SD (n = 3). Unpaired t-test analysis shows a significant difference for the levels of PA 18:1/18:1 (****p < 0.0001), but no significance (n.s.) for PA 18:1/16:1 (p = 0.2576). (C) *In vitro* activity of combined EcPlsY and EcPlsC in presence of a fatty acid mix (oleic acid (FA 18:1) and palmitoleic acid (FA 16:1). As a control no enzyme reactions were performed. Synthesized lipid species were analyzed using LC-MS (table 3). Levels of species were corrected for the internal standard DOPE. Data are mean (average) ± SD (n = 3).

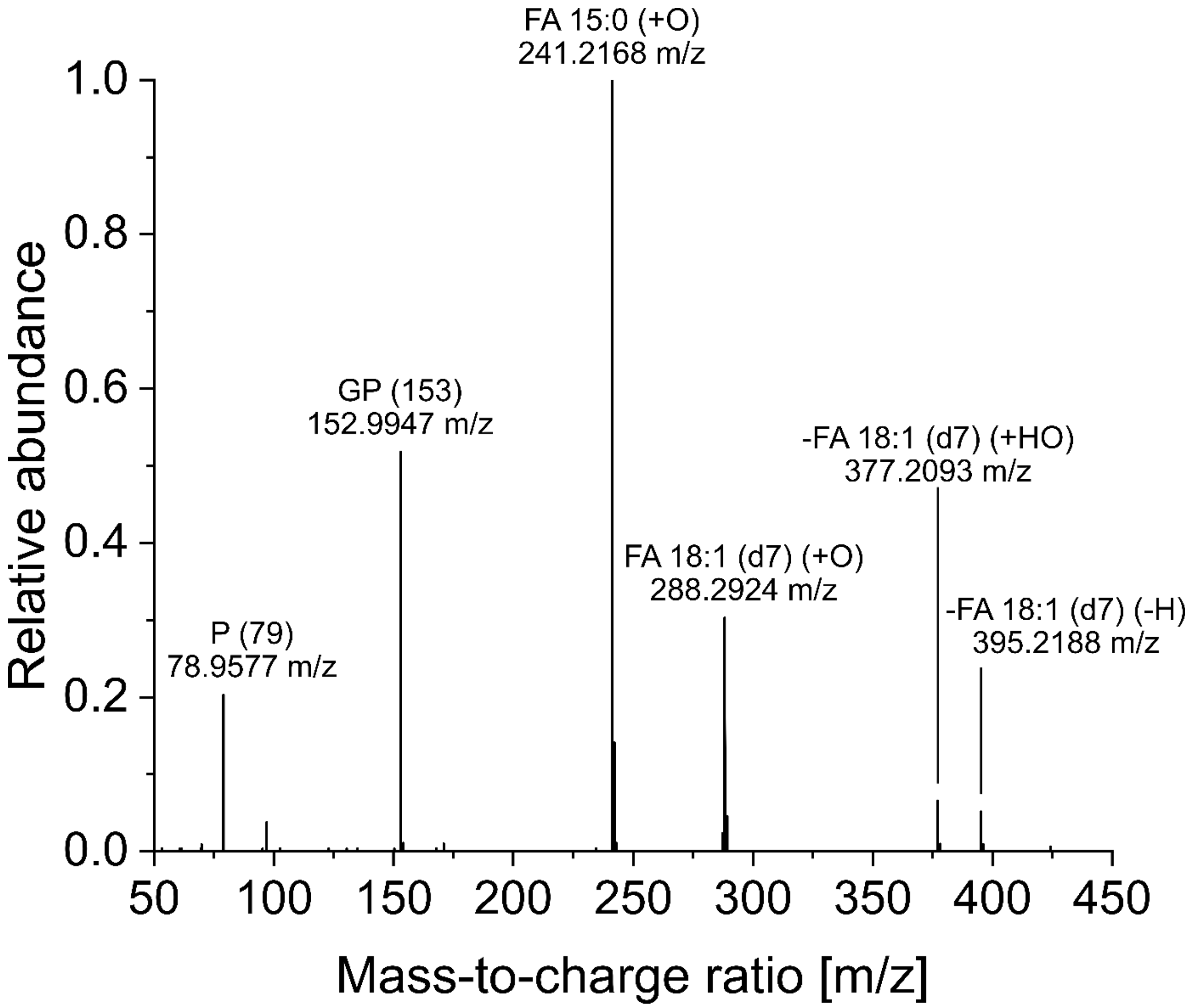

Appendix Figure S3. Fragmentation spectrum of the lipid standard: PA 15:0/18:1(d7). Identified fragments: phosphate (P), glycerol-phosphate (GP), fatty acid *sn*-1 position (FA 15:0 (+O)), fatty acid *sn*-2 position (FA 18:1 (d7) (+O)), Lysophosphatidic acid 15:0 (-FA 18:1(d7) (+HO) and -FA 18:1(d7) (-H)).
