# Supplementary figures and images for: Bacterial lipid synthesizing enzymes PlsY and PlsC utilize both stereo-forms of glycerol-phosphate
Source: EMBO Rep. 2026 Jun 12;27(14):3928–43. doi: 10.1038/s44319-026-00827-z (PMC13400652; doi:10.1038/s44319-026-00827-z)

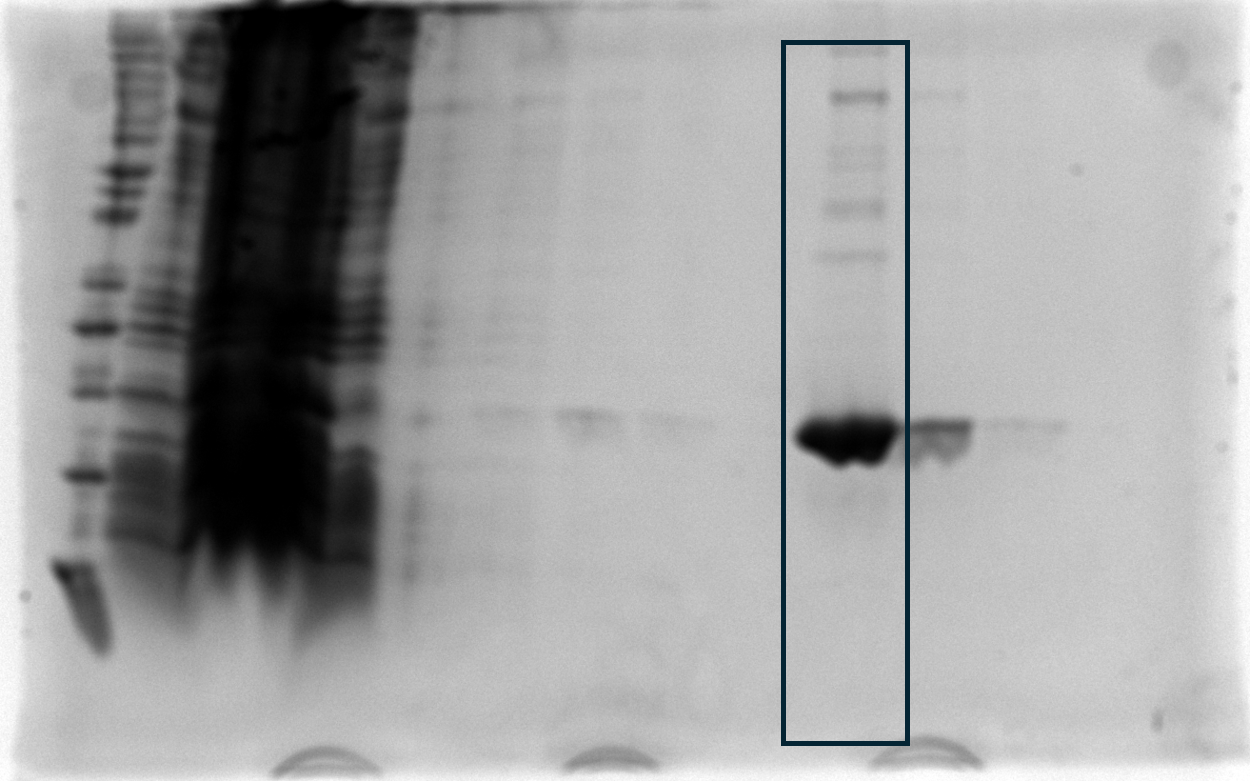

Supplement: Supplementary file 4 — Source data Fig. 2 [file 44319_2026_827_MOESM4_ESM.zip › Figure 2/2B/BsPlsY_Gel_paper.png]

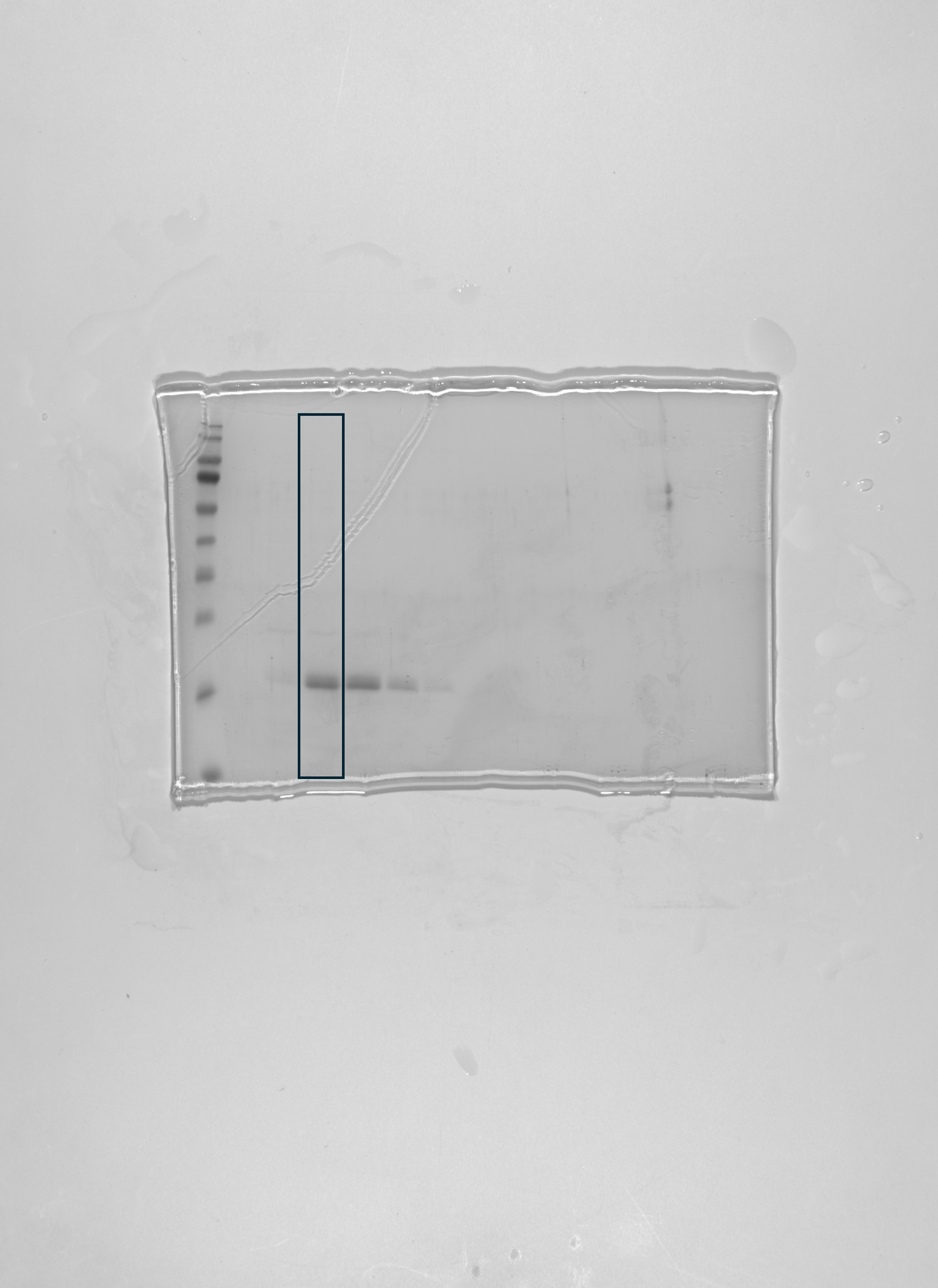

Supplement: Supplementary file 4 — Source data Fig. 2 [file 44319_2026_827_MOESM4_ESM.zip › Figure 2/2B/Gel_EcPlsY_paper.png]

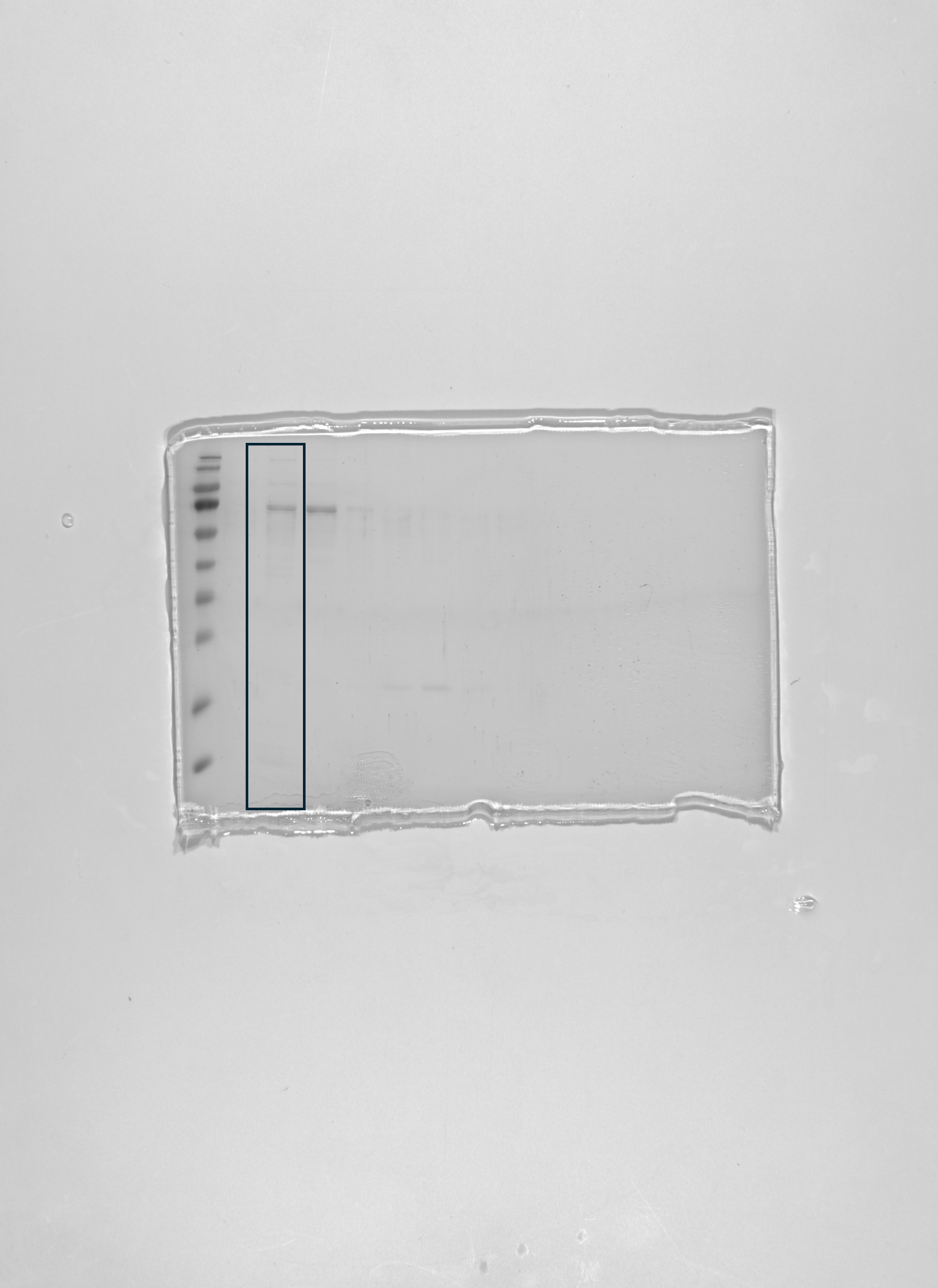

Supplement: Supplementary file 4 — Source data Fig. 2 [file 44319_2026_827_MOESM4_ESM.zip › Figure 2/2B/Gel_FakA_paper.png]

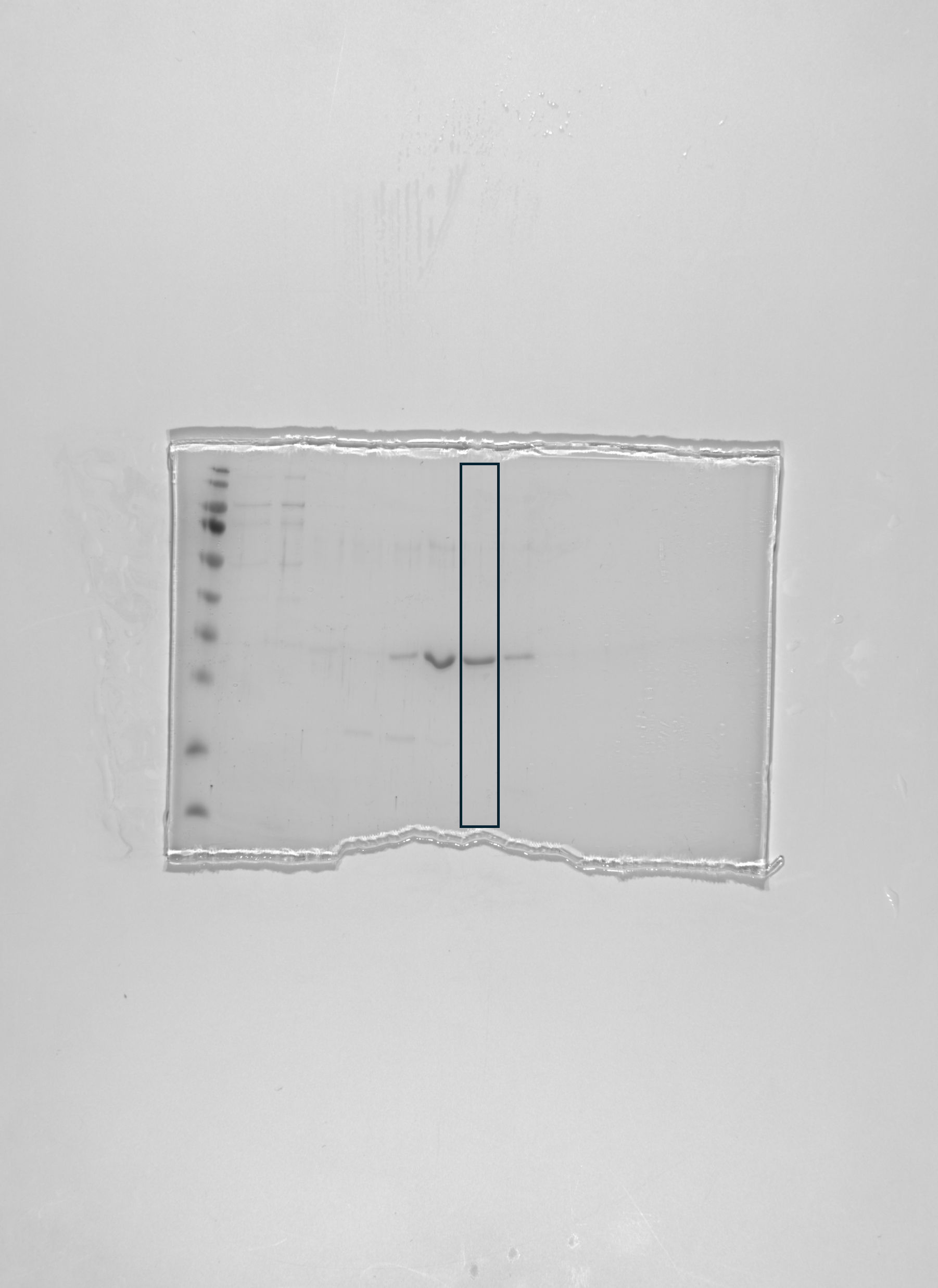

Supplement: Supplementary file 4 — Source data Fig. 2 [file 44319_2026_827_MOESM4_ESM.zip › Figure 2/2B/Gel_FakB_paper.png]

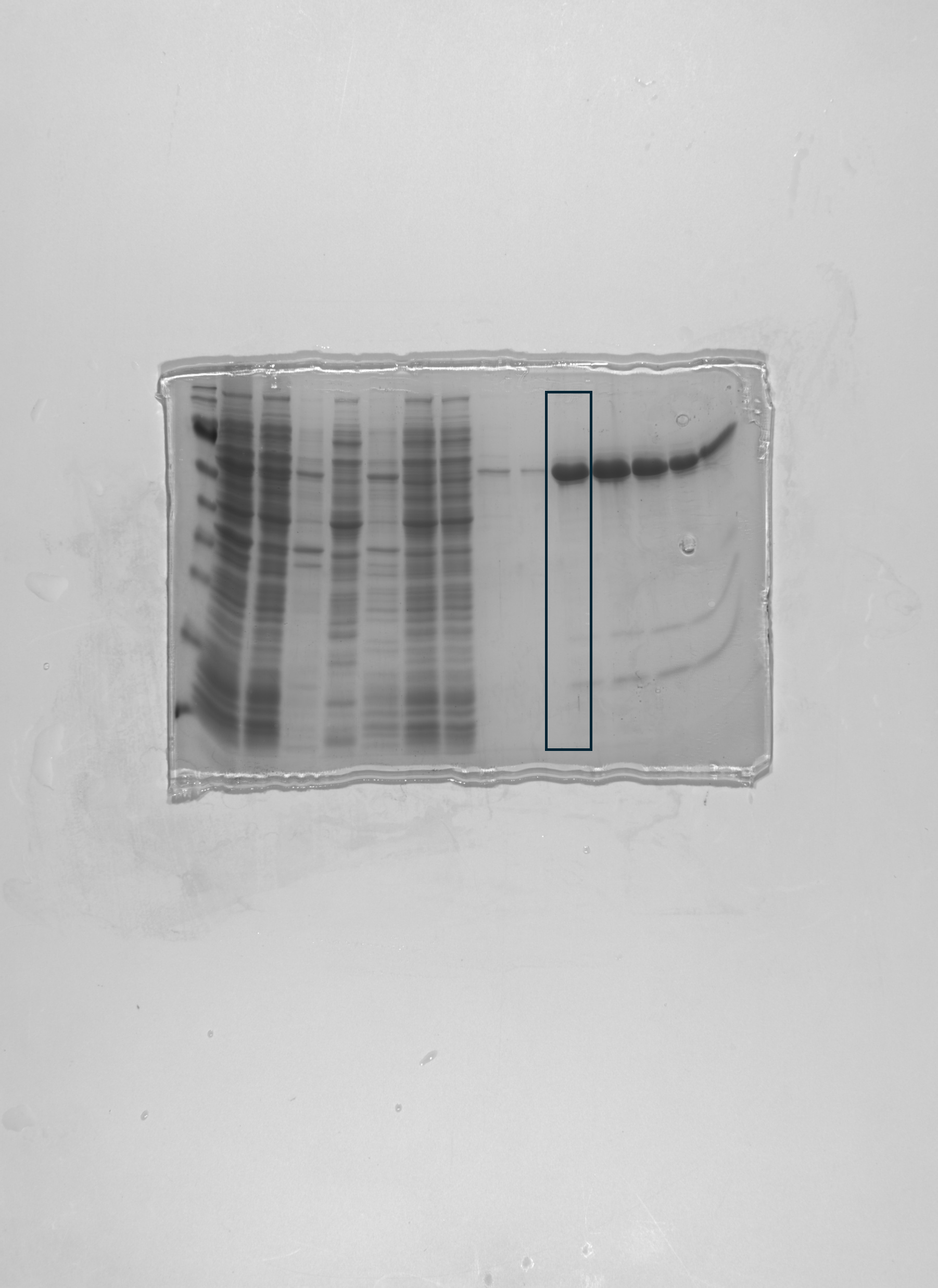

Supplement: Supplementary file 5 — Source data Fig. 3 [file 44319_2026_827_MOESM5_ESM.zip › Figure 3/3B/Gel_FadD_paper.png]

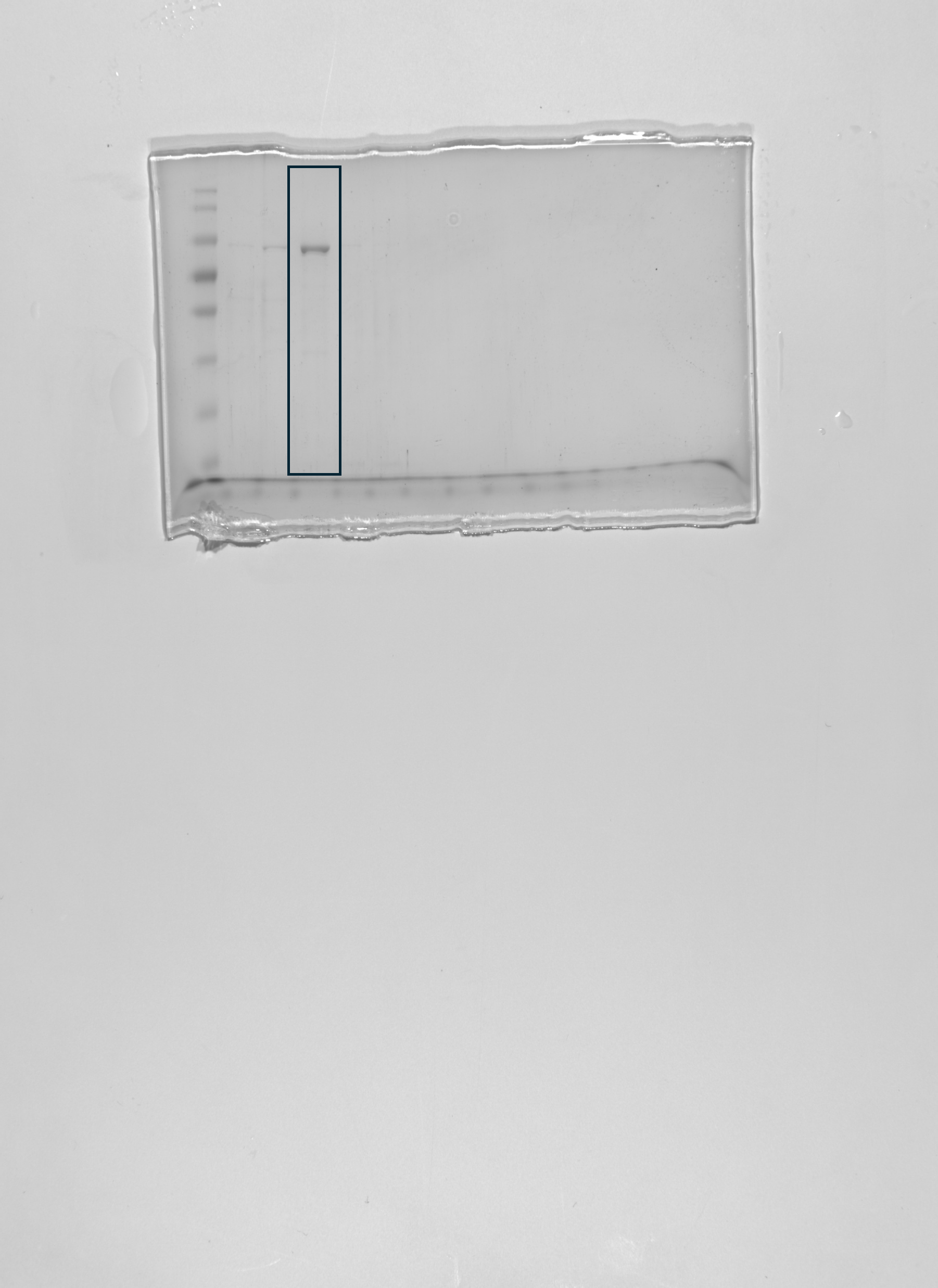

Supplement: Supplementary file 5 — Source data Fig. 3 [file 44319_2026_827_MOESM5_ESM.zip › Figure 3/3B/Gel_PlsB_paper.png]

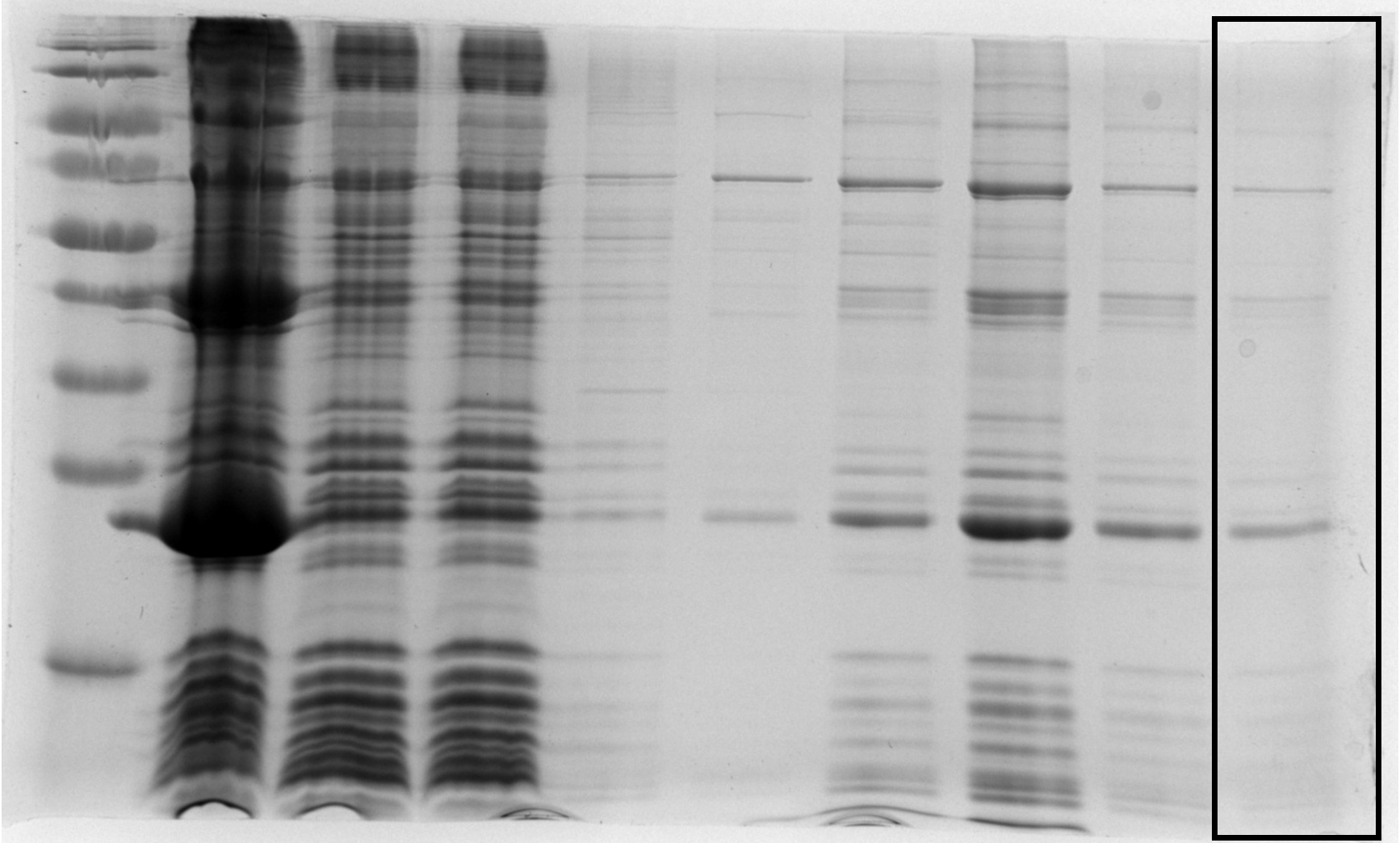

Supplement: Supplementary file 6 — Source data Fig. 4 [file 44319_2026_827_MOESM6_ESM.zip › Figure 4/4A/Gel_Bsubtilis PlsC_paper.png]

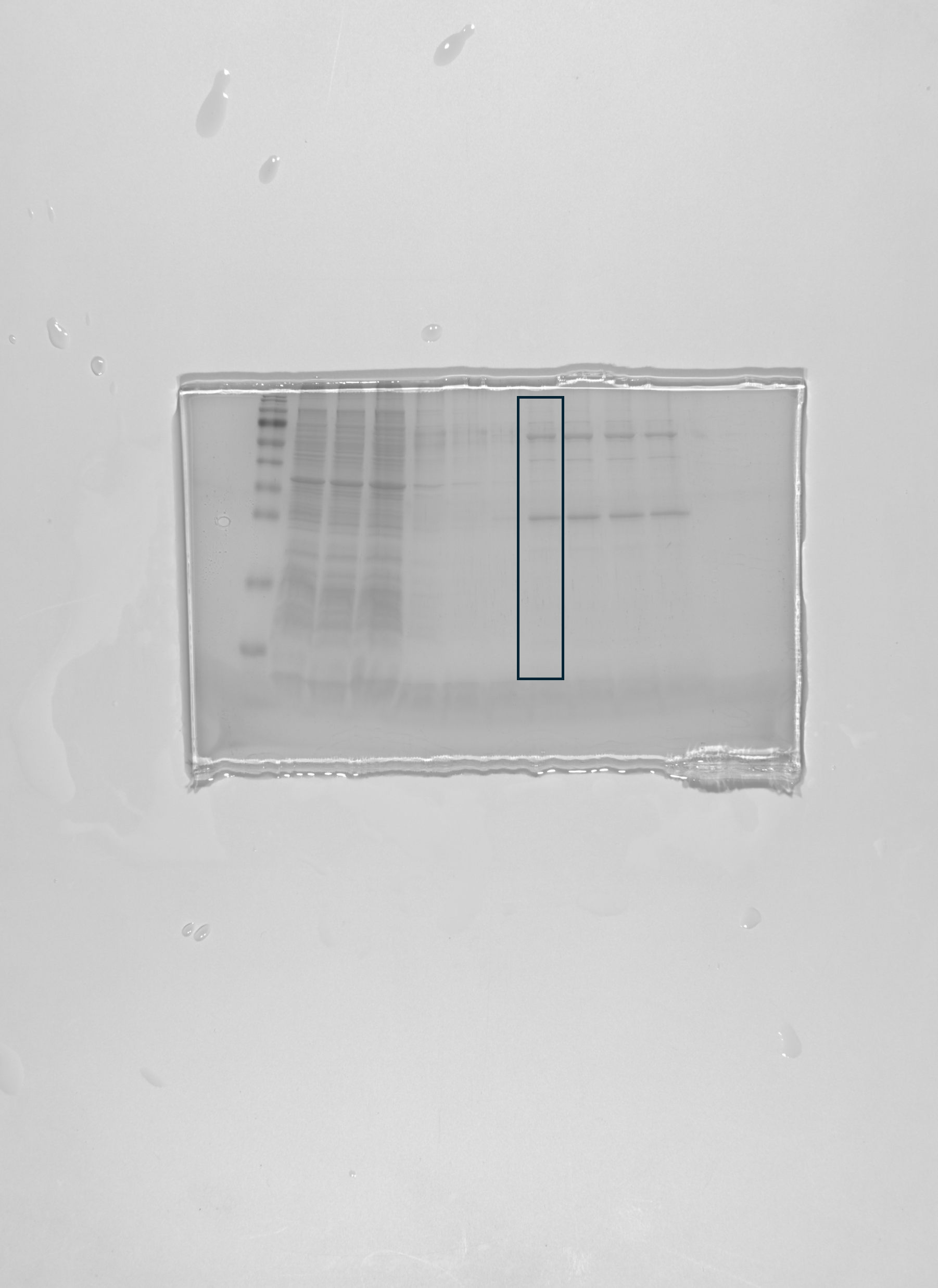

Supplement: Supplementary file 6 — Source data Fig. 4 [file 44319_2026_827_MOESM6_ESM.zip › Figure 4/4A/Gel_EcPlsC_paper.png]
