# Supplementary material for: Bacterial lipid synthesizing enzymes PlsY and PlsC utilize both stereo-forms of glycerol-phosphate
Source: EMBO Rep. 2026 Jun 12;27(14):3928–43. doi: 10.1038/s44319-026-00827-z (PMC13400652; doi:10.1038/s44319-026-00827-z)
Supplement: Supplementary file 2 — Peer Review File [file 44319_2026_827_MOESM2_ESM.pdf]

EMBO reports

Bacterial lipid synthesizing enzymes PlsY and PlsC utilize both stereo-forms of glycerol-phosphate

Philipp Rieche, Mirthe Hoekzema, Sergiy Gan, Greetje A. Berrelkamp-Lahpor, Adanna Ezissi, Marten Exterkate, and Arnold J.M. Driessen

Corresponding author(s): Marten Exterkate (marten.exterkate@hhu.de) , Arnold J.M. Driessen (a.j.m.driessen@rug.nl)

Review Timeline:

Submission Date:	6th Nov 25
Editorial Decision:	17th Dec 25
Revision Received:	27th Feb 26
Editorial Decision:	22nd Apr 26
Revision Received:	18th May 26
Accepted:	22nd May 26

Editor: Martina Rembold

Transaction Report:

(Note: With the exception of the correction of typographical or spelling errors that could be a source of ambiguity, letters and reports are not edited. Depending on transfer agreements, referee reports obtained elsewhere may or may not be included in this compilation. Referee reports are anonymous unless the Referee chooses to sign their reports.)

Dear Dr. Exterkate

Thank you for the submission of your manuscript to EMBO reports. We have now received the full set of referee reports that are all pasted below.

As you will see, the referees acknowledge that the findings are potentially interesting. However, they also have a number of concerns and suggestions how to further strengthen your conclusions that should be addressed.

I would thus like to invite you to revise your manuscript with the understanding that the referee concerns must be fully addressed and their suggestions taken on board. Please address all referee concerns in a complete point-by-point response. Acceptance of the manuscript will depend on a positive outcome of a second round of review. It is EMBO reports policy to allow a single round of major revision only and acceptance or rejection of the manuscript will therefore depend on the completeness of your responses included in the next, final version of the manuscript.

We realize that it is difficult to revise to a specific deadline. In the interest of protecting the conceptual advance provided by the work, we recommend a revision within 3 months (19th Mar 2026). Please discuss the revision progress ahead of this time with the editor if you require more time to complete the revisions.

You can either publish the study as a short report or as a full article. For short reports, the revised manuscript should not exceed 27,000 characters (including spaces but excluding materials & methods and references) and 5 main plus 5 expanded view figures. The results and discussion sections must further be combined, which will help to shorten the manuscript text by eliminating some redundancy that is inevitable when discussing the same experiments twice. For a normal article there are no length limitations, but it should have more than 5 main figures and the results and discussion sections must be separate. In both cases, the entire materials and methods must be included in the main manuscript file.

IMPORTANT NOTE: we perform an initial quality control of all revised manuscripts before re-review. Your manuscript will FAIL this control and the handling will be DELAYED if the following APPLIES:
1) A data availability section providing access to data deposited in public databases is missing. If you have not deposited any data, please add a sentence to the data availability section that explains that.
2) Your manuscript contains statistics and error bars based on n=2. Please use scatter blots in these cases. No statistics should be calculated if n=2.

When submitting your revised manuscript, please review the submission guidelines (https://link.springer.com/journal/44319/submission-guidelines#cms-Revised-submissions) and carefully review the instructions that follow below. Failure to include requested items will delay the evaluation of your revision.

1) a .docx formatted version of the manuscript text (including legends for main figures, EV figures and tables). Please make sure that the changes are highlighted to be clearly visible.

2) individual production quality figure files as .eps, .tif, .jpg (one file per figure). See hhttps://media.springernature.com/original/springer-cms/rest/v1/content/27825798/data/v1 for more info on how to prepare your figures.

3) We replaced Supplementary Information with Expanded View (EV) Figures and Tables that are collapsible/expandable online. A maximum of 5 EV Figures can be typeset. EV Figures should be cited as 'Figure EV1, Figure EV2" etc... in the text and their respective legends should be included in the main text after the legends of regular figures.

- For the figures that you do NOT wish to display as Expanded View figures, they should be bundled together with their legends in a single PDF file called *Appendix*, which should start with a short Table of Content. Appendix figures should be referred to in the main text as: "Appendix Figure S1, Appendix Figure S2" etc. See 'Expanded View' section of the submission guidelines.

- Additional Tables/Datasets should be labeled and referred to as Table EV1, Dataset EV1, etc. Legends have to be provided in a separate tab in case of .xls files. Alternatively, the legend can be supplied as a separate text file (README) and zipped together with the Table/Dataset file.

4) a .docx formatted letter INCLUDING the reviewers' reports and your detailed point-by-point responses to their comments. As part of the EMBO Press transparent editorial process, the point-by-point response is part of the Review Process File (RPF), which will be published alongside your paper.

5) a complete author checklist, which you can download from our author guidelines. Please insert information in the checklist that is also reflected in the manuscript. The completed author checklist will also be part of the RPF.

6) Please note that all corresponding authors are required to supply an ORCID ID for their name upon submission of a revised manuscript (), which can be linked to your profile in the manuscript submission system.

7) Before submitting your revision, primary datasets produced in this study need to be deposited in an appropriate public database. Please remember to provide a reviewer password if the datasets are not yet public. The accession numbers and database should be listed in a formal "Data Availability" section placed after Methods. Please note that the Data Availability Section is restricted to new primary data that are part of this study. * Note - All links should resolve to a page where the data can be accessed. *

If your study has not produced novel datasets, please mention this fact in the Data Availability Section.

8) At EMBO Press we ask authors to provide source data for the main manuscript figures. You will receive a separate email with instructions for providing source data with your revised manuscript, including how to upload and organize the files.

9) Our journal also encourages inclusion of *data citations in the reference list* to directly cite datasets that were re-used and obtained from public databases. Data citations in the article text are distinct from normal bibliographical citations and should directly link to the database records from which the data can be accessed. In the main text, data citations are formatted as follows: "Data ref: Smith et al, 2001" or "Data ref: NCBI Sequence Read Archive PRJNA342805, 2017". In the Reference list, data citations must be labeled with "[DATASET]". A data reference must provide the database name, accession number/identifiers and a resolvable link to the landing page from which the data can be accessed at the end of the reference.

10) Regarding data quantification, the following points must be specified in each figure legend:

- the name of the statistical test used to generate error bars and P values,

- the number (n) of independent experiments (please specify technical or biological replicates) underlying each data point,

- the nature of the bars and error bars (s.d., s.e.m.),

- If the data are obtained from n Program fragment delivered error ``Can't locate object method "less" via package "than" (perhaps you forgot to load "than"?) at //ejpvfs23/sites23b/embor_www/letters/embor_decision_revise_and_review_sr_any.txt line 55.' 2, use scatter blots showing the individual data points.

Discussion of statistical methodology can be reported in the materials and methods section, but figure legends should contain a basic description of n, P and the test applied.

- Please also include scale bars in all microscopy images.

11) The journal requires a statement specifying whether or not authors have competing interests (defined as all potential or actual interests that could be perceived to influence the presentation or interpretation of an article). In case of competing interests, this must be specified in your disclosure statement.

12) All Materials and Methods need to be described in the main text using our 'Structured Methods' format, which is required for all research articles. According to this format, the Methods section includes a Reagents and Tools Table (listing key reagents, experimental models, software and relevant equipment and including their sources and relevant identifiers) followed by a Methods and Protocols section describing the methods using a step-by-step protocol format. The aim is to facilitate adoption of the methodologies across labs. More information on how to adhere to this format as well as a downloadable template (.docx) for the Reagents and Tools Table can be found in our author guidelines.

We would also welcome the submission of cover suggestions, or motifs to be used by our Graphics Illustrator in designing a cover.

As part of the EMBO publication's Transparent Editorial Process, EMBO reports publishes online a Review Process File (RPF) to accompany accepted manuscripts. This File will be published in conjunction with your paper and will include the referee reports, your point-by-point response and all pertinent correspondence relating to the manuscript.

You are able to opt out of this by letting the editorial office know (emboreports@embo.org). If you do opt out, the Review Process File link will point to the following statement: "No Review Process File is available with this article, as the authors have chosen not to make the review process public in this case."

I look forward to seeing a revised form of your manuscript when it is ready.

Yours sincerely,

Martina Rembold, PhD
Senior Editor
EMBO reports

=================

Referee #1:

The so-called lipid divide describes the difference in lipid structure between bacteria and eucaryotes on the on hand and archaea on the other. Bacterial/eucaryal lipids have a glycerol-3-phosphate (G3P) backbone to which fatty acids are esterified. Archaeal lipids differ in the stereochemistry of their backbone, with a glycerol-1-phosphate (G1P) to which isoprenoid tails are connected by ether bonds. However, recent findings have shown that both bacteria and archaea can have lipids with the respective non-typical glycerol enantiomer as well. Within this background, the authors have studied the stereospecificity of the enzymes PlsY, PlsB and PlsC which are involved in the biosynthesis of bacterial phospholipids.

To achieve this, the authors have established an in vitro system to synthesize either G1P- or G3P-based phospholipids which are identified with mass spectrometry. The glycerol-phosphate acyl-transferase (GPAT) PlsY combines glycerol phosphate with Acyl-PO4- which the authors synthesize utilizing the FakA/B complex. The product is lysophosphatidic acid (LPA). PlsY did not show a significant preference between G3P and G1P when tested in buffer with detergent (to solubilize the in vivo membrane-located PlsY).

Some bacteria have an alternative GPAT, PlsB. It combines Acyl-CoA, which is synthesized by FadD, with glycerol phosphate to LPA. PlsB showed a strong preference towards G3P over G1P in the presented experiments.

PlsC, a lyso-phosphatidic acyl transferase (LPAAT), attaches the second acyl chain to LPA using acyl-CoA as acyl donor. This is provided by FadD. To prepare stereospecific LPA, the FakA/B-PlsY reaction described above was integrated. Unlike before, PlsY was reconstituted in liposomes, which led to an apparent preference for G3P, leading to significantly higher (R)-LPA levels than (S)-LPA. The subsequent PlsC reaction can use both (R)-and (S)-LPA. However, due to the unequal availability of those two substrates, no reliable preference could be identified, even though more (R)-PA was produced.

The authors finally use their setup to synthesize specific phosphatidic acids with control over backbone stereospecificity and acyl-chain composition, providing a proof of principle to expand the toolbox available to study more specific membrane properties.

The presented research is original and provides novel insights that the lipid divide is not as strict as previously thought. The experiments are well done, the length of the manuscript is appropriate. For most conclusions drawn, there is strong evidence (exceptions see below). The results are relevant both for the specific field and for a broader audience. I think that the manuscript is suitable for publication in EMBO reports after minor revisions.

Scientific and major remarks:

Line 104: "This enzyme can directly utilize acyl-CoA and acyl-ACP." Figure 1A only shows acyl-CoA as its substrate. This should be made congruent.

Line 124ff, Figure 1B: the text describes an in vitro setup, and in the first experiments the proteins are solubilized by detergent, but the figure shows a membrane. This is confusing at the beginning. I have no perfect idea how to improve this, at least an appropriate sentence in the figure legend would be appropriate.

Line 160ff: there is somehow a break in the information flux. It might be better to describe the purification of the proteins already after the first sentence of the paragraph.

Line 165ff/ Figure 2C/D/Methods: it is unclear whether the sample "FakA/B" contained ATP or not, as the text says "was incubated with FakA/B only in the absence or presence of ATP". Anyway, only one sample is shown with FakA/B. How was the reaction started for this sample? Line 168 suggests that G1/3P was added only with PlsY, whereas the figure legend suggests that G1/3P was also present in the control and FakA/B reactions (which would be better as a control) The figure legend and/or text/Methods part should be improved here.

Fig. 2C/D: Is there an explanation why the FakA/B complex uses less oleic acid in D than in C?

Fig. 2 and others: It would be helpful to mention in the figure legends how the identities of the substances were confirmed. One

could refer to Tab. 3

Line 194: "showed high purity": What about the strong band in the FadD lane (Fig. 3B) at ca. 20 kDa that accounts for at least 25% of the total protein?

Line 214/215: Is PlsB really only present in E. coli or rather in Proteobacteria in general as stated in the introduction?

Line 220: "In contrast, PG from B. subtilis is a mixture of both lipid backbone stereoforms." A reference should be given here. I think the earlier mentioned (13) would be appropriate.

Line 236: "PlsC utilizes both stereo-forms of LPA with different specificity" This ("different specificity") cannot be concluded from the results. Because PlsY reconstituted in liposomes produces more (R)-LPA, there are too many ambiguities in this experiment, and I think this does not allow to declare "different specificity" in the header of the paragraph. As the authors state later (l. 278): "proper kinetic analysis is required". But it is certainly clear that PlsC can use both stereo-forms.
Would it be possible to produce the (S)/(R)-LPA separately in detergent as previously shown (Fig. 2) and then add them to the PlsC reaction in the same amount?

Line 245/Fig. 4A: There is something wrong with the masses: The text says "29 kDa and 55 kDa", but 2x29 is not 55. The Figure shows no bands at 29 but maybe 22 kDa, and one at about 57-60 kDa. BsPlsC shows no band at 55, but at ca. 45 and >70 kDa. This must be clasified. What are the calculated MW of the proteins?

Line 249: there is no data in absence of ATP in fig S1.

Figure 4 C/D: why are there small (but substantial) amounts of PA in the samples without PlsC? This must be clarified, otherwise this calls into question the validity of the assay.

Line 373: "there is no proof of bacterial lipids with isoprenoid chains" This must be changed to "of bacterial G3P-based lipids with isoprenoid chains", because G1P-based ether lipids with isoprenoid chains have been found in bacteria (introduction, ref. 10 and other publications)

Line 422: giving rpm is useless without specifying the rotor/centrifuge

Line 425: The µl Strep-resin information is only informative if the culture volume was specified.

Lines 435 and 448: for how long? It would also be helpful to specify the rotor (at least fixed-angle or swing-out)

Line 439: with the same setting as before?

Line 816/Figure S2: "in presence of oleic acid (FA 18:1) and palmitoleic acid (FA mix)." There is no sample with FA 18:1 alone in C. It also took me some time to realize what "PA 18:1_16:1" is, I only got it when looking at Tab. 3. Maybe one could explain this somewhere in the text (l785ff) or the figure legend.

Figure S2C: I do not understand why the t-test is only shown for PA 18:1/18:1, or why the t-test was made at all (there should be no products in the control reaction)? What do we learn here?

Minor remarks (language, smaller errors, ...):

Line 16: "consist of" instead of "exist of"

Line 57: "only recently": I think, the cited references (6) from 1977 and (7) from 1997 are not "recently" any more.

Line 61: "consists of" or "contains"

Line 66f: "... possess genes for synthesis of G3P, among which GlpA and GlpD which have G3PDH-like activity": mix-up of genes and protein names

Line 91: "question", not "questions"

Line 92f: "mechanisms ... remain unclear"

Line 100: abbreviation GPAT is only introduced in line 114

Line 114f: ", from the Gram-positive Bacillus subtilis, ": I think the 2 commata should be removed, they disturb the flux of reading

Line 159: remove "by"

Line 243: some word is missing, e.g. "to achieve optimal function"

Line 252: missing reference to Fig. S1C?

Line 257: The "these" is too much here

Figure 5A: Oleoyl-CoA, not "Coa"

Line 376: "striking", not "strikingly"

Table 1, line 2 and 3: b. subtilis, capitalize B. and italicize organism

Line 414: "indicate", not "indicating"

Line 436 and 450: better "resuspended" than "solved"

Line 527-529: words after comma/colon sometimes capitalized, sometimes not

Line 614: this was published in 1978, not 1977

Line 746: better "consists of", not "exists out of"

Line 771 set "in vitro" in italics

Line 816: EcPlsY

Referee #2:

Rieche et al. conducted a series of elaborated experiments to address the substrate specificity of bacterial lipid biosynthesis and demonstrated that some bacterial systems are intrinsically capable of accommodating two different stereoisomers and thus is stereochemically more flexible than previously thought. The methodology is sound and the presented data are informative. I support the publication of this interesting piece of work, while I also recommend some modifications mainly about the manuscript organization and evolutionary implications.

1) Introduction
a) The paragraph seems to mix arguments about the stereochemistry in different carbon positions. It is not clear why the stereochemistry of the PG headgroup has something to do with that of glycerol phosphate (G1P/G3P) (L58-64). The authors may suggest that two PG stereoisomers with an opposite configuration in the headgroup (sn-1', sn-3') must be biosynthesized from different precursors with an opposite configuration to each other (i.e. G3P and G1P). However, in the cited reference 7, the stereochemistry of glycerol phosphate (R-configuration) is identical for all detected PG isomers and the difference resides only in the carbon in the headgroup. Please clarify this point, if the co-presence of G1P and G3P is really the case.
b) I expected that the paragraph L56-L72 is about the chirality surrounding G1P/G3P from the first sentence. However, L64-L72 is about the taxonomic distribution of G1P/G3P and is not directly related to chirality. This latter part may be separated as a different paragraph, focusing on the taxonomic and evolutionary analyses of lipid biosynthesis genes. In this regard, why are some relevant previous works not included? E.g. Villanueva et al., The ISME Journal (2021) 15:168-182, Bridging the membrane lipid divide: bacteria of the FCB group superphylum have the potential to synthesize archaeal ether lipids.
c) Much of the introduction in the current manuscript is about G1P/G3P and head groups (hydrophilic region), but they are largely omitted in Fig. 1 and also not discussed in later sections that are all about fatty acyl chain attachments after G1P/G3P formation. Hence, this is also confusing. In fact, the novelty of this work is that the authors investigated the stereo-specificity for the latter part of lipid biosynthesis after G1P/G3P, which were previously not studied in detail.
d) In the introduction (particularly L37-L88), a lot of enzymes are introduced without full spellings and visual aids and thus it is hard to follow for non-experts. It is recommended to let the readers get the whole picture of phospholipid biosynthesis in bacteria/eukaryotes (and perhaps also archaea). I think Fig. 1A should be expanded for this purpose (Fig. 1B may be separated as a different figure). The lipid heterochirality should be articulated in the figure so that the readers can quickly understand where the issue is. Also, at least the (simplified) chemical structures of key enzymatic products should be shown. Additionally, the difference in lipid biosynthesis in gram-positive and gram-negative bacteria also needs to be articulated - i.e. presence/absence of PlsB.
It would also be helpful to suggest the presence of the AraM pathway and the unknown bacterial pathway for G1P biosynthesis in parallel for a better understanding of later sections, but this may not be mandatory.

2) Evolutionary implications
a) Bacillus species seem to have unique features in terms of membrane lipid biosynthesis. They possess AraM for G1P biosynthesis. They also have PcrB to prenylate G1P with C35 isoprenoid chains (Guldan et al., 2011, as also cited in the manuscript). C35 isoprenoids are in fact rare in bacteria (Ueda et al., 2015). In Bacillus, C35 chains are also utilized for uncommon respiratory quinones and pentacyclic baciterpenol (Sato et al., 2011). There is currently no consensus about why Bacillus produces such 'weird' lipids. I was just wondering if the authors have any ideas or thoughts on this topic, which might enrich the discussions and prospects of this manuscript.
Guldan et al., Functional assignment of an enzyme that catalyzes the synthesis of an archaea-type ether lipid in bacteria. Angew. Chem. Int. Ed. 50, 8188-8191 (2011).
Ueda et al., Biosynthesis of Sesterterpenes, Head-to-Tail Triterpenes, and Sesquarterpenes in Bacillus clausii: Identification of Multifunctional Enzymes and Analysis of Isoprenoid Metabolites. ChemBioChem 16:1371-7 (2015).
Sato et al., Sesquarterpenes (C35 terpenes) biosynthesized via the cyclization of a linear C35 isoprenoid by a tetraprenyl-β-curcumene synthase and a tetraprenyl-β-curcumene cyclase: identification of a new terpene cyclase. J. Am. Chem. Soc. 133:9734-7 (2011).
b) I am just curious about if there is any attempt to perform a similar experiment for archaeal membrane lipid biosynthesis, in order to see the chiral flexibility for the archaeal membrane?
c) If the authors intend to argue for the potential wide distribution of G1P and G3P-based heterochiral membranes in bacteria, it would be helpful to make a summary table for the taxonomic distribution of putative G1P-based lipids biosynthesis that is investigated so far in bacteria, including the species in this manuscript and from previous studies (if such a table has not been published elsewhere).

Specific comments
L67: The meaning is unclear - 'among which GlpA and GlpD which have G3PDH-like activity'
L67: GlpA, GlpD and G3PDH need to be spelled out as this is the first introduction. Ditto for many other enzymes throughout the manuscript.
L71: The meaning is unclear - 'the precise function if the pathway is still unknown'
L87: In reference 17, crenarchaeol/GDGT isomers seem to be both derived from the G1P conformation. Also, the cited paper does not argue for the co-presence of G1P and G3P, as far as I understand.
L135: PL needs to be spelled out.
L220: A reference needed for PG in B. subtilis.
L300: Lipid tail incorporation is not shown in Fig. 1B, even though it is shown in Fig. 5B. All other reactions during lipid biosynthesis are included in Fig. 1B. Hence, please include lipid tail incorporation in Fig. 1B, too.
L474: Purchase info for G1P and G3P seems to be missing.
Figs. 1A&1B: The arrow between LPA and PA passes over PlsC, unlike other arrows that do not cross enzyme blocks. This is confusing because it looks like that PA biosynthesis can skip PlsC.
Fig. 1B: Part of the figure reappears in Figs. 2A, 3A, 4B and 5A and this is confusing, even though I understand that they are shown for clarification purposes. For example, how about emphasizing individual subregions of Fig. 1B by adding a square frame with a numbering so that the readers can understand the relationship between Fig. 1B and subsequent figures?
Figs. 2-5: The pictures of key enzymatic products would be helpful.

Referee #3:

The study by Rieche & Hoekzema et al. represents an important step towards understanding the origin of the lipid stereochemical divide between bacteria and archaea, or rather, further supports the emerging opinion that the lipid divide is less strict than commonly thought. Specifically, the authors experimentally tested the stereospecificity of the bacterial lipid biosynthetic enzymes PlsB, PlsC, and PlsY, finding that PlsY and PlsC but not PlsB can utilize both stereochemistries, i.e., the G1P (archaeal) vs. G3P (bacterial) configurations. The experimental design is elegant and the results and conclusions are convincing. I have only very minor comments pertaining to the mass spectrometry results, as detailed below.

Table 3: Please also list retention times and diagnostic fragments used for identification.

Figure 2: Use „." As decimal separator for p values in caption.

Figure 5: Change axis label for spectra: they do not show mass but mass-to-charge ratio. Include the exact masses of the fragments in additioon to the 16:1/18:1 labels. The corresponding lyso fragment(s) should also be shown as further proof. The peaks are tailing strongly or contain a co-eluting compound. Can the authors confirm that the MS/MS spectrum is identical throughout the peak?

Rebuttal

Referee #1:

The so-called lipid divide describes the difference in lipid structure between bacteria and eucaryotes on the on hand and archaea on the other. Bacterial/eucaryal lipids have a glycerol-3-phosphate (G3P) backbone to which fatty acids are esterified. Archaeal lipids differ in the stereochemistry of their backbone, with a glycerol-1-phosphate (G1P) to which isoprenoid tails are connected by ether bonds. However, recent findings have shown that both bacteria and archaea can have lipids with the respective non-typical glycerol enantiomer as well. Within this background, the authors have studied the stereospecificity of the enzymes PlsY, PlsB and PlsC which are involved in the biosynthesis of bacterial phospholipids.

To achieve this, the authors have established an in vitro system to synthesize either G1P- or G3P-based phospholipids which are identified with mass spectrometry. The glycerol-phosphate acyl-transferase (GPAT) PlsY combines glycerol phosphate with Acyl-PO4- which the authors synthesize utilizing the FakA/B complex. The product is lysophosphatidic acid (LPA). PlsY did not show a significant preference between G3P and G1P when tested in buffer with detergent (to solubilize the in vivo membrane-located PlsY).

Some bacteria have an alternative GPAT, PlsB. It combines Acyl-CoA, which is synthesized by FadD, with glycerol phosphate to LPA. PlsB showed a strong preference towards G3P over G1P in the presented experiments.

PlsC, a lyso-phosphatidic acyl transferase (LPAAT), attaches the second acyl chain to LPA using acyl-CoA as acyl donor. This is provided by FadD. To prepare stereospecific LPA, the FakA/B-PlsY reaction described above was integrated. Unlike before, PlsY was reconstituted in liposomes, which led to an apparent preference for G3P, leading to significantly higher (R)-LPA levels than (S)-LPA. The subsequent PlsC reaction can use both (R)-and (S)-LPA. However, due to the unequal availability of those two substrates, no reliable preference could be identified, even though more (R)-PA was produced.

The authors finally use their setup to synthesize specific phosphatidic acids with control over backbone stereospecificity and acyl-chain composition, providing a proof of principle to expand the toolbox available to study more specific membrane properties.

The presented research is original and provides novel insights that the lipid divide is not as strict as previously thought. The experiments are well done, the length of the manuscript is appropriate. For most conclusions drawn, there is strong evidence (exceptions see below). The results are relevant both for the specific field and for a broader audience. I think that the manuscript is suitable for publication in EMBO reports after minor revisions.

Scientific and major remarks:

Line 104: "This enzyme can directly utilize acyl-CoA and acyl-ACP." Figure 1A only shows acyl-CoA as its substrate. This should be made congruent.

Correct, we added an additional arrow from Acyl-ACP to PlsB in figure 1A.

Line 124ff, Figure 1B: the text describes an in vitro setup, and in the first experiments the proteins are solubilized by detergent, but the figure shows a membrane. This is confusing at the beginning. I have no perfect idea how to improve this, at least an appropriate sentence in the figure legend would be appropriate.

The idea for figure 1B was to introduce the FadD and FakA/B, but we agree with the referee that the current representation is confusing. As for each of the experiments (Figure 2-5) we already provide schematic overviews separately, we decided to remove Figure 1B in its current form and just show simple reaction schemes for FakA/B and FadD (now Figure 1C).

Line 160ff: there is somehow a break in the information flux. It might be better to describe the purification of the proteins already after the first sentence of the paragraph.

We agree and moved the part describing the purification of the proteins upward. Additionally, small changes were made to maintain textual accuracy and flow.

Line 165ff/ Figure 2C/D/Methods: it is unclear whether the sample "FakA/B" contained ATP or not, as the text says "was incubated with FakA/B only in the absence or presence of ATP". Anyway, only one sample is shown with FakA/B. How was the reaction started for this sample? Line 168 suggests that G1/3P was added only with PlsY, whereas the figure legend suggests that G1/3P was also present in the control and FakA/B reactions (which would be better as a control) The figure legend and/or text/Methods part should be improved here.

The samples FakA/B contained ATP and the FakA/B sample was started by addition of ATP. Textual changes have been made. G1P or G3P was present in all samples. During initial testing, the FakA/B reaction was executed in the presence of G1P and G3P, showing no background production of LPA. To avoid waste of the rather expensive G1P, we added G3P to all control and FakA/B reactions. To clarify this in the manuscript we made additional changes.

Fig. 2C/D: Is there an explanation why the FakA/B complex uses less oleic acid in D than in C?

We also noticed this peculiar observation. One difference between the two samples is the amount of starting Oleic acid (0.6mM in Figure 2C and 0.5mM in Figure 2D), but this should not have any impact. We also used three different enzyme batches, but the handling (growth, expression, purification etc.) was the same. As the main goal of the FakA/B reaction is to produce acyl-Pi, and importantly, we did not observe any background formation of LPA, we did not further look into this, as the focus of the experiment was on the activity of PlsY.

Fig. 2 and others: It would be helpful to mention in the figure legends how the identities of the substances were confirmed. One could refer to Tab. 3

References to table 3 have been added to Figures 2-5, S1 and S2. Also, by suggestion of referee 3, table 3 has been extended with a list of the retention times and diagnostic fragments used for identification.

Line 194: "showed high purity": What about the strong band in the FadD lane (Fig. 3B) at ca. 20 kDa that accounts for at least 25% of the total protein?
Correct. We performed a western blot analysis by using an anti-strep antibody (Figure R1), suggesting that this is not a degradation product, but an impurity. We took another look at the other FadD purifications used for the experiments and found that the impurity was greatly reduced. Regardless, this had no influence on the activity of FadD, nor on the other enzymes in coupled assays, so we replaced the SDS-PAGE in the manuscript.

Figure for referee with unpublished data and its description has been removed upon request by the authors.

Line 214/215: Is PlsB really only present in E. coli or rather in Proteobacteria in general as stated in the introduction?

In a review article by Zhang et al. [1], a phylogenetic distribution of PlsB and PlsX/Y is presented. There PlsB is widely present in alfa, beta, gamma and delta proteobacteria. Moreover, PlsB has been found in some actinobacteria. We meant here that *B. subtilis* has no PlsB, but we see now that this was poorly phrased and understand the confusion. Textual changes have been made.

Line 220: "In contrast, PG from B. subtilis is a mixture of both lipid backbone stereoforms." A reference should be given here. I think the earlier mentioned (13) would be appropriate.

Reference is added.

Line 236: "PlsC utilizes both stereo-forms of LPA with different specificity" This ("different specificity") cannot be concluded from the results. Because PlsY reconstituted in liposomes produces more (R)-LPA, there are too many ambiguities in this experiment, and I think this does not allow to declare "different specificity" in the header of the paragraph. As the authors state later (l. 278): "proper kinetic analysis is required". But it is certainly clear that PlsC can use both stereo-forms. Would it be possible to produce the (S)/(R)-LPA separately in detergent as previously shown (Fig. 2) and then add them to the PlsC reaction in the same amount?

This is a valid point, and indeed there are too many ambiguities in these experiments to make this point. We therefore removed the “with different specificity” statement from the header of the paragraph.
The proposed experiment, regarding the production of (S)/(R)-LPA separately in detergent, is a good suggestion. However, the presence of the detergent is not compatible with the liposomal setup needed for PlsC activity, as it will dissolve the liposomes, thereby actively blocking PlsC activity. Hence this setup is not possible.

Line 245/Fig. 4A: There is something wrong with the masses: The text says "29 kDa and 55 kDa", but 2x29 is not 55. The Figure shows no bands at 29 but maybe 22 kDa, and one at about 57-60 kDa. BsPlsC shows no band at 55, but at ca. 45 and >70 kDa. This must be clasified. What are the calculated MW of the proteins?

The calculated MW weight for EcPlsC is 28.9 kDa and for BsPlsC 22.7 kDa. Hence the band for BsPlsC runs at the correct height. In the case of EcPlsC, the protein indeed runs at a lower MW on SDS-PAGE, slightly below 25kDa. This is typically observed for integral membrane proteins that bind more SDS than soluble proteins. EcPlsC is membrane anchored through two-helices rich in hydrophobic and aromatic residues. We figure that this unusual hydrophobic region also binds more SDS than commonly observed for soluble proteins. Further, in another study in which the in vitro transcription translation of EcPlsC was performed also observes a mass slightly below 25kDa [2]. To further confirm the MW of EcPlsC, western blot analysis was performed by using an anti-strep antibody (Figure R2). Indeed, a clear dimer is observed for EcPlsC slightly above 55 kDa.

Figure for referee with unpublished data and its description has been removed upon request by the authors.

Line 249: there is no data in absence of ATP in fig S1.

Correct, we replaced this by: "the control reaction"

Figure 4 C/D: why are there small (but substantial) amounts of PA in the samples without PlsC? This must be clarified, otherwise this calls into question the validity of the assay.

Indeed, we observe small amounts of PA in the samples with PlsY, but without PlsC. We suspect a small amount of background synthesis is caused by minor amounts of co-purified PlsC. In a different research project, we study the possibility of complex formation between phospholipid synthesizing enzymes. Strikingly, we could show via pull-down interactions studies with purified protein that PlsY and PlsC indeed interact with each other, suggesting that co-purification is likely (Figure R3). We are currently in the process of publishing these results: https://papers.ssrn.com/sol3/papers.cfm?abstract_id=6022921

Despite the suspected minor amounts of co-purified PlsC present in the PlsY samples, addition of PlsC clearly shows a large increase in produced PA. By subtracting the background PA, we would basically end up with the same results. As this does not interfere with our main observation, that PlsC can utilize both stereoforms of LPA, we accepted the minor PA background levels.

Figure for referee with unpublished data and its description has been removed upon request by the authors.

Line 373: "there is no proof of bacterial lipids with isoprenoid chains" This must be changed to "of bacterial G3P-based lipids with isoprenoid chains", because G1P-based ether lipids with isoprenoid chains have been found in bacteria (introduction, ref. 10 and other publications)

Correct, we have made the replacement.

Line 422: giving rpm is useless without specifying the rotor/centrifuge

We converted all ‘rpm’ to ‘xg’.

Line 425: The µl Strep-resin information is only informative if the culture volume was specified.

Correct, we added the culture volume.

Lines 435 and 448: for how long? It would also be helpful to specify the rotor (at least fixed-angle or swing-out).

Time and Rotor type were added (fixed angle).

Line 439: with the same setting as before?

Correct the information has been added to the materials and methods.

Line 816/Figure S2: "in presence of oleic acid (FA 18:1) and palmitoleic acid (FA mix)." There is no sample with FA 18:1 alone in C. It also took me some time to realize what "PA 18:1_16:1" is, I only got it when looking at Tab. 3. Maybe one could explain this somewhere in the text (l785ff) or the figure legend.

Correct, we meant to state that the FA mix consisted of oleic acid and palmitoleic acid. We made the textual adjustments.
We further explained the meaning of “PA 18:1_16:1” directly in the text.

Figure S2C: I do not understand why the t-test is only shown for PA 18:1/18:1, or why the t-test was made at all (there should be no products in the control reaction)? What do we learn here?

We wanted to show that trace amounts of PA 18:1/18:1 are produced in the enzymatic reaction with PlsY and PlsC, which is not that apparent from the graph itself. However, we agree with the referee that the t-test is obsolete, so we removed this. We further clarified this in the manuscript text.

Minor remarks (language, smaller errors, ...):

Line 16: "consist of" instead of "exist of"
corrected

Line 57: "only recently": I think, the cited references (6) from 1977 and (7) from 1997 are not "recently" any more.
Correct, we removed this and rephrased.

Line 61: "consists of" or "contains"
corrected to “consist of”

Line 66f: "... possess genes for synthesis of G3P, among which GlpA and GlpD which have G3PDH-like activity": mix-up of genes and protein names
corrected

Line 91: "question", not "questions"
corrected

Line 92f: "mechanisms ... remain unclear"
corrected

Line 100: abbreviation GPAT is only introduced in line 114
corrected

Line 114f: ", from the Gram-positive Bacillus subtilis, ": I think the 2 commata should be removed, they disturb the flux of reading
removed

Line 159: remove "by"
removed

Line 243: some word is missing, e.g. "to achieve optimal function"
corrected

Line 252: missing reference to Fig. S1C?
corrected

Line 257: The "these" is too much here
removed

Figure 5A: Oleoyl-CoA, not "Coa"
Figure adjusted

Line 376: "striking", not "strikingly"
corrected

Table 1, line 2 and 3: b. subtilis, capitalize B. and italicize organism
corrected

Line 414: "indicate", not "indicating"
corrected

Line 436 and 450: better "resuspended" than "solved"
adjusted

Line 527-529: words after comma/colon sometimes capitalized, sometimes not
adjusted

Line 614: this was published in 1978, not 1977

Line 746: better "consists of", not "exists out of"
corrected

Line 771 set "in vitro" in italics
done

Line 816: EcPlsY
done

Referee #2:

Rieche et al. conducted a series of elaborated experiments to address the substrate specificity of bacterial lipid biosynthesis and demonstrated that some bacterial systems are intrinsically capable of accommodating two different stereoisomers and thus is stereochemically more flexible than previously thought. The methodology is sound and the presented data are informative. I support the publication of this interesting piece of work, while I also recommend some modifications mainly about the manuscript organization and evolutionary implications.

1) Introduction
a) The paragraph seems to mix arguments about the stereochemistry in different carbon positions. It is not clear why the stereochemistry of the PG headgroup has something to do with that of glycerol phosphate (G1P/G3P) (L58-64). The authors may suggest that two PG stereoisomers with an opposite configuration in the headgroup (sn-1', sn-3') must be biosynthesized from different precursors with an opposite configuration to each other (i.e. G3P and G1P). However, in the cited reference 7, the stereochemistry of glycerol phosphate (R-configuration) is identical for all detected PG isomers and the difference resides only in the carbon in the headgroup. Please clarify this point, if the co-presence of G1P and G3P is really the case.

The first 3 points (1a-c) are about the stereochemistry of the headgroup (specifically of PG), vs. the stereochemistry of the glycerol-phosphate lipid backbone. The referee rightfully points out that our presented results are purely focused on the stereochemistry of the glycerol-phosphate lipid backbone. In the introduction we mentioned the stereochemistry of the headgroup mainly to point out that both G3P and G1P are found in bacteria. For a long time, the presence of G1P in bacteria was not considered as no G1P synthase was identified. However, we do see how in the current form this might come across as confusing and partially irrelevant as the rest of the manuscript solely focuses on the stereochemistry of the lipid backbone. We therefore removed the statements regarding the chirality of the headgroup.

b) I expected that the paragraph L56-L72 is about the chirality surrounding G1P/G3P from the first sentence. However, L64-L72 is about the taxonomic distribution of G1P/G3P and is not directly related to chirality. This latter part may be separated as a different paragraph, focusing on the taxonomic and evolutionary analyses of lipid biosynthesis genes. In this regard, why are some relevant previous works not included? E.g. Villanueva et al., The ISME Journal (2021) 15:168-182, Bridging the membrane lipid divide: bacteria of the FCB group superphylum have the potential to synthesize archaeal ether lipids.

We would like to thank the referee for pointing this out and included the provided reference.

c) Much of the introduction in the current manuscript is about G1P/G3P and head groups (hydrophilic region), but they are largely omitted in Fig. 1 and also not discussed in later sections that are all about fatty acyl chain attachments after G1P/G3P formation. Hence, this is also confusing. In fact, the novelty of this work is that the authors investigated the stereo-specificity for the latter part of lipid biosynthesis after G1P/G3P, which were previously not studied in detail.

See response to comment 1a.

d) In the introduction (particularly L37-L88), a lot of enzymes are introduced without full spellings and visual aids and thus it is hard to follow for non-experts. It is recommended to let the readers get the whole picture of phospholipid biosynthesis in bacteria/eukaryotes (and perhaps also archaea). I think Fig. 1A should be expanded for this purpose (Fig. 1B may be separated as a different figure). The lipid heterochirality should be articulated in the figure so that the readers can quickly understand where the issue is. Also, at least the (simplified) chemical structures of key enzymatic products should be shown. Additionally, the difference in lipid biosynthesis in gram-positive and gram-negative bacteria also needs to be articulated - i.e. presence/absence of PlsB.
It would also be helpful to suggest the presence of the AraM pathway and the unknown bacterial pathway for G1P biosynthesis in parallel for a better understanding of later sections, but this may not be mandatory.

We have introduced the missing full spellings and added additional visual aids to figure 1. In short, heterochirality of glycerol-phosphate and LPA is shown by adding chemical structures.
We also added additional information regarding the presence absence of PlsB. It should be noted though that there are gram-negative bacteria that do not contain PlsB. According to Zhang et al (JLR, 2008) [1] PlsB is mostly limited to proteobacteria and some actinobacteria. We therefore chose to follow this classification.

2) Evolutionary implications
a) Bacillus species seem to have unique features in terms of membrane lipid biosynthesis. They possess AraM for G1P biosynthesis. They also have PcrB to prenylate G1P with C35 isoprenoid chains (Guldan et al., 2011, as also cited in the manuscript). C35 isoprenoids are in fact rare in bacteria (Ueda et al., 2015). In Bacillus, C35 chains are also utilized for uncommon respiratory quinones and pentacyclic baciterpenol (Sato et al., 2011). There is currently no consensus about why Bacillus produces such 'weird' lipids. I was just wondering if the authors have any ideas or thoughts on this topic, which might enrich the discussions and prospects of this manuscript.
Guldan et al., Functional assignment of an enzyme that catalyzes the synthesis of an archaea-type ether lipid in bacteria. Angew. Chem. Int. Ed. 50, 8188-8191 (2011).
Ueda et al., Biosynthesis of Sesterterpenes, Head-to-Tail Triterpenes, and Sesquarterpenes in Bacillus clausii: Identification of Multifunctional Enzymes and Analysis of Isoprenoid Metabolites. ChemBioChem 16:1371-7 (2015).
Sato et al., Sesquarterpenes (C35 terpenes) biosynthesized via the cyclization of a linear C35 isoprenoid by a tetraprenyl-β-curcumene synthase and a tetraprenyl-β-curcumene cyclase: identification of a new terpene cyclase. J. Am. Chem. Soc. 133:9734-7 (2011).

Indeed, it is entirely unclear why Bacillus makes these special lipids, and we are also intrigued by the topic. As our data focuses on the Bacillus enzymes PlsY and PlsC, and we do not have any further information on the AraM or PcrB, we decided to not discuss this further in the article, as it would be pure speculation from our side.

b) I am just curious about if there is any attempt to perform a similar experiment for archaeal membrane lipid biosynthesis, in order to see the chiral flexibility for the archaeal membrane?

Previously data on the production of archaeal phospholipids in *E. coli* employing the archaeal key enzymes GGGPS and DGGGPS provided only evidence (as assessed by NMR) for the production of

G1P-based ether lipids, whereas *E. coli* readily produces G3P [3]. Noteworthy, direct enzyme tests of the key enzyme GGGPS show it can utilize G1P as well as G3P as substrate. Interesting follow-up experiments would involve various bacterial and archaeal acyl-transferases to get a better understanding on the promiscuity towards chirality in both prokaryotic domains of life.

c) If the authors intend to argue for the potential wide distribution of G1P and G3P-based heterochiral membranes in bacteria, it would be helpful to make a summary table for the taxonomic distribution of putative G1P-based lipids biosynthesis that is investigated so far in bacteria, including the species in this manuscript and from previous studies (if such a table has not been published elsewhere).

G1P-based lipid biosynthesis has so far only been studied in some Bacilli and thermotolerant bacteria, where their presence has been observed by mass spectrometry and other analytical techniques by Palyzová et al. (Řezanka group) [4,5]. So far, such studies have really been limited, and at this stage a table would not really add much simply by lack of completeness. Indeed, we suggest a potential wider distribution of G1P-based lipids in bacteria, but this will only be the case if these bacteria contain the PlsX/Y system and produce G1P simultaneously. We made some additional changes in the text refining the requirements for a wide distribution heterochiral membranes

Specific comments
L67: The meaning is unclear - 'among which GlpA and GlpD which have G3PDH-like activity'
This was phrased oddly by us. GlpA and GlpD are actual G3PDH's. We adjusted this in the text.

L67: GlpA, GlpD and G3PDH need to be spelled out as this is the first introduction. Ditto for many other enzymes throughout the manuscript.
We went through the manuscript and spelled out all enzymes upon first mention.

L71: The meaning is unclear - 'the precise function if the pathway is still unknown'
We meant the function of the produced lipids. We therefore replaced this by "the cellular function of these lipids is still unknown"

L87: In reference 17, crenarchaeol/GDGT isomers seem to be both derived from the G1P conformation. Also, the cited paper does not argue for the co-presence of G1P and G3P, as far as I understand.
This is correct. We misinterpreted the stated information. As it is no longer relevant for this manuscript, we removed the text.

L135: PL needs to be spelled out.
corrected

L220: A reference needed for PG in B. subtilis.
corrected

L300: Lipid tail incorporation is not shown in Fig. 1B, even though it is shown in Fig. 5B. All other reactions during lipid biosynthesis are included in Fig. 1B. Hence, please include lipid tail incorporation in Fig. 1B, too.
Due to comments of other referees, we removed Figure 1B.

L474: Purchase info for G1P and G3P seems to be missing.
The information has been added

Figs. 1A&1B: The arrow between LPA and PA passes over PlsC, unlike other arrows that do not cross enzyme blocks. This is confusing because it looks like that PA biosynthesis can skip PlsC.
Fig. 1B: Part of the figure reappears in Figs. 2A, 3A, 4B and 5A and this is confusing, even though I understand that they are shown for clarification purposes. For example, how about emphasizing individual subregions of Fig. 1B by adding a square frame with a numbering so that the readers can understand the relationship between Fig. 1B and subsequent figures?
Other Referees also found Figure 1B confusing, so it has been removed.

Figs. 2-5: The pictures of key enzymatic products would be helpful.
We have added the chemical structures of the two glycerol-phosphates, LPAs and PAs in figure 1. To not overcrowd the experimental figures 2-5, we did not add these chemical structures again.

Referee #3:

The study by Rieche & Hoekzema et al. represents an important step towards understanding the origin of the lipid stereochemical divide between bacteria and archaea, or rather, further supports the emerging opinion that the lipid divide is less strict than commonly thought. Specifically, the authors experimentally tested the stereospecificity of the bacterial lipid biosynthetic enzymes PlsB, PlsC, and PlsY, finding that PlsY and PlsC but not PlsB can utilize both stereochemistries, i.e., the G1P (archaeal) vs. G3P (bacterial) configurations. The experimental design is elegant and the results and conclusions are convincing. I have only very minor comments pertaining to the mass spectrometry results, as detailed below.

Table 3: Please also list retention times and diagnostic fragments used for identification.

All have been added.

Figure 2: Use „." As decimal separator for p values in caption.

Corrected. All other figure captions were checked and adjusted as well.

Figure 5: Change axis label for spectra: they do not show mass but mass-to-charge ratio. Include the exact masses of the fragments in addition to the 16:1/18:1 labels. The corresponding lyso fragment(s) should also be shown as further proof. The peaks are tailing strongly or contain a co-eluting compound. Can the authors confirm that the MS/MS spectrum is identical throughout the peak?

Axis label was adjusted. Exact masses were added. Furthermore, the lyso-fragment(s) were added. The MS/MS spectrum is identical throughout the peak (ratio between fragments remains the same). The observed peak tailing is caused by the LC-chromatography setup. We observe this for all our free-phosphate lipids (LPA, PA), including standards.

Furthermore, the lyso-fragment(s) were added. While doing this we made a surprising observation. Assuming that the *sn*-2 FA fragment (FA *sn*-2 (+O)) is dominant, we expected the *sn*-1 LPA fragments (-FA *sn*-2 (+HO) and -FA *sn*-2 (-H)) to be dominant. However, in both cases we observed that the *sn*-2 LPA fragments (-FA *sn*-1 (+HO) and -FA *sn*-1 (-H)) were dominant, which is contradictory. We then checked again the raw MS data files corresponding to the samples in figure 5 and realized we mixed up our chromatograms. To clarify this, we checked a PA standard (PA 15:0/18:1(d7)) and observed that with our MS settings the *sn*-1 FA fragment (FA *sn*-1 (+O)) is dominant over the *sn*-2 FA fragment (FA *sn*-2 (+O)) (fig. S3). Likewise, we analyzed the fragmentation pattern of the in figure S1 synthesized PA 18:1/16:1 from the substrates LPA 18:1 and palmitoleoyl-CoA (synthesized from FA 16:1 via FadD). The fragmentation pattern (Figure R4) is coherent with the (PA 15:0/18:1(d7)) fragmentation, confirming our observations.

We corrected this in the manuscript and added the PA standard (15:0/18:1(d7) PA) exemplary chromatogram in the supplementary.

Figure for referee with unpublished data and its description has been removed upon request by the authors.

References:

(1) Zhang, Y. M.; Rock, C. O. Thematic Review Series: Glycerolipids. Acyltransferases in Bacterial Glycerophospholipid Synthesis. *J. Lipid Res.* **2008**, *49* (9), 1867–1874. https://doi.org/10.1194/jlr.R800005-JLR200 [doi].

(2) Scott, A.; Noga, M. J.; de Graaf, P.; Westerlaken, I.; Yildirim, E.; Danelon, C. Cell-Free Phospholipid Biosynthesis by Gene-Encoded Enzymes Reconstituted in Liposomes. *PLoS One* **2016**, *11* (10), e0163058. https://doi.org/10.1371/journal.pone.0163058.

(3) Caforio, A.; Siliakus, M. F.; Exterkate, M.; Jain, S.; Jumde, V. R.; Andringa, R. L. H.; Kengen, S. W. M.; Minnaard, A. J.; Driessen, A. J. M.; van der Oost, J. Converting Escherichia Coli into an Archaebacterium with a Hybrid Heterochiral Membrane. *Proc. Natl. Acad. Sci. U. S. A.* **2018**, *115* (14). https://doi.org/10.1073/pnas.1721604115.

(4) Palyzová, A.; Guschina, I. A.; Řezanka, T. Chiral Analysis of Glycerol Phosphates - Can Bacteria Biosynthesize Heterochiral Phospholipid Membranes? *J. Chromatogr. A* **2022**, *1676*, 463267. https://doi.org/https://doi.org/10.1016/j.chroma.2022.463267.

(5) Palyzová, A.; Šmrhová, T.; Kapinusová, G.; Škrob, Z.; Uhlík, O.; Řezanka, T. Stereochemistry of Phosphatidylglycerols from Thermotolerant Bacteria Isolated Thermal Springs. *J. Chromatogr. A* **2025**, *1739*, 465517. https://doi.org/https://doi.org/10.1016/j.chroma.2024.465517.

Dear Marten,

Thank you for the submission of your revised manuscript to EMBO reports. We have now received the reports from the referees that were asked to assess it (copied below). Referee #3 only filled the summary table recommendation without further commenting.

As you will see, both referees are very positive about the study and request only minor changes to clarify text and figures.

From the editorial side, there are also a few things that we need before we can proceed with the official acceptance of your study.

- There is a discrepancy in an author name: Greetje A. Berrelkamp-Lahpor in the manuscript vs. Greetje Berrelkam in the online submission system. Please resolve this.

- The manuscript sections should be in the following order: Title page - Abstract & Keywords - Introduction - Results - Discussion - Methods - Data Availability - Acknowledgments - Disclosure Statement & Competing Interests - References - Figure Legends - Main Tables with legends - Expanded View Figure Legends.

- Abbreviations section needs to be removed from the manuscript. Abbreviations should be defined in brackets after their first mention in the text, not in a list of abbreviations.

- Author Information should be removed from the manuscript.

- Please remove the figures from the manuscript file. The legends should stay at the end of the manuscript.

- Please update the 'Conflict of interest' paragraph to 'Disclosure and competing interests statement'.

- Regarding the Author Contributions, we now use CRediT to specify the contributions of each author in the journal submission system. CRediT replaces the author contribution section, which therefore needs to be removed from the manuscript text. You can use the free text box in our system if you wish to provide more detailed descriptions. See also guide to authors https://www.embopress.org/page/journal/14693178/authorguide#authorshipguidelines.

- The references need to be alphabetical, not numerical; et al needs to be used after 10 author names.

- The Appendix needs page numbers - in the Table of Contents and throughout the file.

- The short text in the Appendix uses wrong callouts, e.g., to fig. S1B instead of Appendix Fig. S1B.

- The Reagents and Tools table uploaded as separate document is sufficient. Our typesetters will add it to the Methods section. Therefore, please remove the R&T table from the Methods secion.

- Data availability: please update the missing link to BioStudies, as discussed. We also ask to deposit raw mass spectrometry data to a relevant public repository and you indicated that you were looking into this issue.

- Source data: Please upload each Figure folder as a separate zip file.

- Doing a spot check on the source data I noticed that the gel in Figure 2B, BsPlsY has been quite narrowly cropped (on the left side of the band).

- Please address the following points in the figure legends:
A) Please note that figure titles for figures 1, 2 are not provided, This needs to be rectified.
B) Please define the annotated p values ****/***/**/* as well as provide the exact p-values for the same in the legend of figure S2 C as appropriate.
C) Please provide the exact p values in the legends of figures 3C, 4C, S1 D, S2 A, B, unless p<0,0001.
D) Please indicate the statistical test used for data analysis in the legend of figure S2 C

- Your response to the referees contains unpublished data in form of reviewer figures and a link to unpublished data. We publish the referee reports and your response to these alongside the paper as Peer Review File. Please let us know in case you want to remove any of the reviewer figures. Please note that all data in the Peer Review File counts as published and that you would have to cite this document were your to re-use these in future publications.

- As a standard procedure we edit title and abstract of manuscripts. In this case, the title is too long. Our cutoff is 100 characters

incl. spaces. I have shortened it to 124 characters but ask you to see whether you can shorten it further. Please also review the changes to the abstract. I added a bit more general background for the general readership of our journals. Feel free to edit this further.

- Finally, EMBO Reports papers are accompanied online by
A) a short (1-2 sentences) summary of the findings and their significance,
B) 2-3 bullet points highlighting key results and
C) a schematic summary figure that provides a sketch of the major findings (not a data image).
Please provide the summary figure as a separate file in PNG or JPG format at a size of 550x300 pixels (width x height). Please note that the size is rather small and that text needs to be readable at the final size. Please send us this information along with the revised manuscript.

We look forward to seeing a final version of your manuscript as soon as possible.

With kind regards,

Martina

Martina Rembold, PhD
Senior Editor
EMBO reports

==================

Referee #1:

The authors have responded to all my remarks and made appropriate changes in the manuscript.
There are only some minor errors that should be corrected before publication:
Line 60: G3PDH abbreviation is not introduced. See also l 60f, where it is done fine for G1PDH.
Line 114: Missing points in B. subtilis and E. coli
Line 210: missing space ("phosphate as")
Line 213: Capitalize Unpaired
Line 299 and 304; Figs. 4C/D: It should perhaps be mentioned in the figure that the control reaction contained (I assume) G3P
Line 398: what does "destruction" mean here? Better: "extraction"?
Line 463: In the rebuttal letter, the authors said they used the same settings for centrifugation as before and now added this to the text, but the settings are not added here.
Line 473: centrifugation time is still missing
Fig. S2C: t-test has been removed from the legend text, as outlined in the rebuttal letter, but is still marked in the figure (with the *)

Referee #3
[supports publication without revision]

==============================

Bacterial phospholipid synthesizing enzymes PlsY and PlsC utilize both stereo-forms of the glycerol-phosphate lipid backbone

The chemical composition of membrane lipids differs between eukarya, bacteria and archaea. The central dogma posits that the stereochemistry of phospholipids in bacteria is distinct from archaea. Bacterial phospholipids consist of fatty acid lipid tails esterified to the sn-glycerol 3-phosphate lipid backbone (G3P), whereas archaeal phospholipids comprise isoprenoid lipid tails ether-linked to the stereochemical different sn-glycerol 1-phosphate (G1P). This segregation, the "lipid divide", is however not as strict as previously thought. Recent reports demonstrate that both glycerol-phosphate backbones are present in phospholipids from various Gram-positive bacteria. To test if the stereochemical variability can be attributed to conventional lipid biosynthesis, we characterize the stereospecificity of the relevant glycerol-phosphate acyl transferases PlsY and PlsB, as well as the lysophosphatidic acid acyl transferase PlsC, catalyzing the key steps in phospholipid biosynthesis yielding phosphatidic acid, both in the Gram-positive B. subtilis and the Gram-negative E. coli. While PlsB is strictly stereospecific for glycerol 3-phosphate, PlsY and PlsC can utilize both stereo-forms of the glycerol-phosphate. Hence, the variability in lipid backbone stereochemistry is

an intrinsic part of bacterial phospholipid biogenesis, questioning the supposedly strict stereochemical segregation of bacteria and archaea after the lipid divide.

Rebuttal

Referee #1:

The authors have responded to all my remarks and made appropriate changes in the manuscript. There are only some minor errors that should be corrected before publication:

Line 60: G3PDH abbreviation is not introduced. See also l 60f, where it is done fine for G1PDH.

- Changed to: “glycerol-3-phosphate dehydrogenase (G3PDH)” and “glycerol-1-phosphate dehydrogenase (G1PDH).

Line 114: Missing points in B. subtilis and E. coli

- The dots have been added.

Line 210: missing space ("phosphate as")

- changed

Line 213: Capitalize Unpaired

- changed

Line 299 and 304; Figs. 4C/D: It should perhaps be mentioned in the figure that the control reaction contained (I assume) G3P

- This is now added to the figure legend.

Line 398: what does "destruction" mean here? Better: "extraction"?

- What is meant is the chemical processing of the lipids to enable analysis via the specific MS-analysis technique, which includes ‘destruction’ (or break-down) of the lipids through derivatization. We have now changed the text to: “…but the lipid chemical processing and analysis method used might have overlooked bacterial ester-bonded phospholipids.”

Line 463: In the rebuttal letter, the authors said they used the same settings for centrifugation as before and now added this to the text, but the settings are not added here.

- It seems like we looked over a few individual settings. We corrected this now. In all cases the centrifuge speed (in xg) and time have been noted down, as well as the rotor type and centrifuge used.

Line 473: centrifugation time is still missing

- See comment above

Fig. S2C: t-test has been removed from the legend text, as outlined in the rebuttal letter, but is still marked in the figure (with the *)

- The symbol has been removed from the figure.

Editorial comments:

There is a discrepancy in an author name: Greetje A. Berrelkamp-Lahpor in the manuscript vs. Greetje Berrelkam in the online submission system. Please resolve this.

- Done

The manuscript sections should be in the following order: Title page - Abstract & Keywords - Introduction - Results - Discussion - Methods - Data Availability - Acknowledgments - Disclosure Statement & Competing Interests - References - Figure Legends - Main Tables with legends - Expanded View Figure Legends.

- Done

Abbreviations section needs to be removed from the manuscript. Abbreviations should be defined in brackets after their first mention in the text, not in a list of abbreviations.

- Done

Author Information should be removed from the manuscript.

- Done

Please remove the figures from the manuscript file. The legends should stay at the end of the manuscript.

- Done

Please update the 'Conflict of interest' paragraph to 'Disclosure and competing interests statement'.

- Done

Regarding the Author Contributions, we now use CRediT to specify the contributions of each author in the journal submission system. CRediT replaces the author contribution section, which therefore needs to be removed from the manuscript text. You can use the free text box in our system if you wish to provide more detailed descriptions. See also guide to authors https://www.embopress.org/page/journal/14693178/authorguide#authorshipguidelines.

- Done

The references need to be alphabetical, not numerical; et al needs to be used after 10 author names.

- Done

The Appendix needs page numbers - in the Table of Contents and throughout the file.

- Done

The short text in the Appendix uses wrong callouts, e.g., to fig. S1B instead of Appendix Fig. S1B.

- Done

The Reagents and Tools table uploaded as separate document is sufficient. Our typesetters will add it to the Methods section. Therefore, please remove the R&T table from the Methods section.

- Done

Data availability: please update the missing link to BioStudies, as discussed. We also ask to deposit raw mass spectrometry data to a relevant public repository and you indicated that you were looking into this issue.

- Done

Source data: Please upload each Figure folder as a separate zip file.

- Done

Doing a spot check on the source data I noticed that the gel in Figure 2B, BsPlsY has been quite narrowly cropped (on the left side of the band).

- The cropping of the gel in figure 2B has been loosened.

Please address the following points in the figure legends:
A) Please note that figure titles for figures 1, 2 are not provided, This needs to be rectified.
B) Please define the annotated p values ****/***/**/* as well as provide the exact p-values for the same in the legend of figure S2 C as appropriate.
C) Please provide the exact p values in the legends of figures 3C, 4C, S1 D, S2 A, B, unless p<0,0001.
D) Please indicate the statistical test used for data analysis in the legend of figure S2 C

- A) Done.

- B) One referee did not see the added value of the statistics, so it has been completely removed now.
- C) Exact P values have been provided in all annotated figure legends.
- D) Not applicable any longer (see comment at B).

Your response to the referees contains unpublished data in form of reviewer figures and a link to unpublished data. We publish the referee reports and your response to these alongside the paper as Peer Review File. Please let us know in case you want to remove any of the reviewer figures. Please note that all data in the Peer Review File counts as published and that you would have to cite this document were your to re-use these in future publications.

- Please remove all reviewer figures from the Peer Review File.

As a standard procedure we edit title and abstract of manuscripts. In this case, the title is too long. Our cutoff is 100 characters incl. spaces. I have shortened it to 124 characters but ask you to see whether you can shorten it further. Please also review the changes to the abstract. I added a bit more general background for the general readership of our journals. Feel free to edit this further.

- Bacterial lipid synthesizing enzymes PlsY and PlsC utilize both stereo-forms of glycerol-phosphate

Finally, EMBO Reports papers are accompanied online by
A) a short (1-2 sentences) summary of the findings and their significance,
B) 2-3 bullet points highlighting key results and
C) a schematic summary figure that provides a sketch of the major findings (not a data image).
Please provide the summary figure as a separate file in PNG or JPG format at a size of 550x300 pixels (width x height). Please note that the size is rather small and that text needs to be readable at the final size. Please send us this information along with the revised manuscript.

- A): For decades, it was thought that bacterial and archaeal membrane lipids not only differ in chemical structure, but also in the stereochemical organization of the glycerol-phosphate backbone. This phenomenon, also known as the lipid divide, must have occurred during early evolution. However, recent insights demonstrate that both stereochemical configurations are present in the phospholipids from various Gram-positive bacteria. Here, we demonstrate that the ubiquitously present acyltransferases PlsY and PlsC can utilize both glycerol-phosphate stereo-forms, suggesting that backbone variability is an intrinsic feature of bacterial phospholipid biogenesis rather than an anomaly.

- B):
    - Present ubiquitously in bacteria, the acyltransferases PlsY and PlsC utilize both stereo-forms of glycerol-phosphate.
    - The acyltranferase PlsB is stereospecific for glycerol-3-phosphate

    - Backbone heterochirality of phospholipids observed in some bacteria can be attributed to PlsY activity.

- C): schematic summary figure uploaded.

2nd Revision - Editorial Decision 22nd May 2026

Dr. Marten Exterkate
Heinrich Heine University Düsseldorf
Membrane Biogenesis and Lipidomics
Universitätsstraße 1
Gebäude: 26.44 Etage/Raum: 01.070
Düsseldorf, Nordrhein-Westfalen 40225
Germany

Dear Marten,

Thank you for implementing the final minor changes. I am very pleased to accept your manuscript for publication in the next available issue of EMBO reports. Thank you for your contribution to our journal.

Your manuscript will be processed for publication by EMBO Press. It will be copy edited and you will receive page proofs prior to publication. Please note that you will be contacted by Springer Nature Author Services to complete licensing and payment information.

You may qualify for financial assistance for your publication charges - either via a Springer Nature fully open access agreement or an EMBO initiative. Check your eligibility: https://link.springer.com/journal/44319/how-to-publish-with-us

Should you be planning a Press Release on your article, please notify the production team via this form in order to coordinate publication and release dates: https://forms.cloud.microsoft/e/eH4A8JVP6g

If you have any questions, please do not hesitate to contact the Editorial Office. Thank you for your contribution to EMBO Reports.

Kind regards,

Martina

Martina Rembold, PhD
Senior Editor
EMBO reports

>>> Please note that it is EMBO Reports policy for the transcript of the editorial process (containing referee reports and your response letter) to be published as an online supplement to each paper. If you do NOT want this, you will need to inform the Editorial Office via email immediately. More information is available here: https://link.springer.com/partners/embo-press/editorial-policies#Peer%20review

EMBO Press Author Checklist

Corresponding Author Name: Marten Exterkate	
Journal Submitted to: EMBO Reports	
Manuscript Number: EMBOR-2025-63129V1	

USEFUL LINKS FOR COMPLETING THIS FORM
The EMBO Journal - Author Guidelines
EMBO Reports - Author Guidelines
Molecular Systems Biology - Author Guidelines
EMBO Molecular Medicine - Author Guidelines

Reporting Checklist for Life Science Articles (updated January
This checklist is adapted from Materials Design Analysis Reporting (MDAR) Checklist for Authors. MDAR establishes a minimum set of requirements in transparent reporting in the life sciences (see Statement of Task: 10.31222/osf.io/9sm4x). Please follow the journal's guidelines in preparing your
Please note that a copy of this checklist will be published alongside your article.

Abridged guidelines for figures
1. Data
The data shown in figures should satisfy the following conditions:
- the data were obtained and processed according to the field's best practice and are presented to reflect the results of the experiments in an accurate and unbiased manner.
- ideally, figure panels should include only measurements that are directly comparable to each other and obtained with the same assay.
- plots include clearly labeled error bars for independent experiments and sample sizes. Unless justified, error bars should not be shown for technical
- if n<5, the individual data points from each experiment should be plotted. Any statistical test employed should be justified.
- Source Data should be included to report the data underlying figures according to the guidelines set out in the authorship guidelines on Data

2. Captions
Each figure caption should contain the following information, for each panel where they are relevant:
- a specification of the experimental system investigated (eg cell line, species name).
- the assay(s) and method(s) used to carry out the reported observations and measurements.
- an explicit mention of the biological and chemical entity(ies) that are being measured.
- an explicit mention of the biological and chemical entity(ies) that are altered/varied/perturbed in a controlled manner.
- the exact sample size (n) for each experimental group/condition, given as a number, not a range;
- a description of the sample collection allowing the reader to understand whether the samples represent technical or biological replicates (including how many animals, litters, cultures, etc.).
- a statement of how many times the experiment shown was independently replicated in the laboratory.
- definitions of statistical methods and measures:
  - common tests, such as t-test (please specify whether paired vs. unpaired), simple χ2 tests, Wilcoxon and Mann-Whitney tests, can be unambiguously identified by name only, but more complex techniques should be described in the methods section;
  - are tests one-sided or two-sided?
  - are there adjustments for multiple comparisons?
  - exact statistical test results, e.g., P values = x but not P values < x;
  - definition of 'center values' as median or average;
  - definition of error bars as s.d. or s.e.m.

Please complete ALL of the questions below.
Select "Not Applicable" only when the requested information is not relevant for your study.

Materials

Newly Created Materials	Information included in the manuscript?	In which section is the information available? (Reagents and Tools Table, Materials and Methods, Figures, Data Availability Section)
New materials and reagents need to be available; do any restrictions apply?	Yes	Reagents and Tools table and Materials and Methods

Antibodies	Information included in the manuscript?	In which section is the information available? (Reagents and Tools Table, Materials and Methods, Figures, Data Availability Section)
For **antibodies** provide the following information:<br>- Commercial antibodies: RRID (if possible) or supplier name, catalogue number and or/clone number<br>- Non-commercial: RRID or citation	Not Applicable	

DNA and RNA sequences	Information included in the manuscript?	In which section is the information available? (Reagents and Tools Table, Materials and Methods, Figures, Data Availability Section)
**Short novel DNA or RNA including primers, probes**: provide the sequences.	Yes	Reagents and Tools table and Materials and Methods

Cell materials	Information included in the manuscript?	In which section is the information available? (Reagents and Tools Table, Materials and Methods, Figures, Data Availability Section)
**Cell lines:** Provide species information, strain. Provide accession number in repository **OR** supplier name, catalog number, clone number, and/**OR** RRID.	Not Applicable	
**Primary cultures:** Provide species, strain, sex of origin, genetic modification status.	Not Applicable	
Report if the cell lines were recently **authenticated** (e.g., by STR profiling) and tested for mycoplasma contamination.	Not Applicable	

Experimental animals	Information included in the manuscript?	In which section is the information available? (Reagents and Tools Table, Materials and Methods, Figures, Data Availability Section)
**Laboratory animals or Model organisms:** Provide species, strain, sex, age, genetic modification status. Provide accession number in repository **OR** supplier name, catalog number, clone number, **OR** RRID.	Not Applicable	
**Animal observed in or captured from the field:** Provide species, sex, and age where possible.	Not Applicable	
Please detail **housing and husbandry conditions**.	Not Applicable	

Plants and microbes	Information included in the manuscript?	In which section is the information available? (Reagents and Tools Table, Materials and Methods, Figures, Data Availability Section)
**Plants:** provide species and strain, ecotype and cultivar where relevant, unique accession number if available, and source (including location for collected wild specimens).	Not Applicable	
**Microbes:** provide species and strain, unique accession number if available, and source.	Yes	Reagents and Tools Table and Materials and Methods

Human research participants	Information included in the manuscript?	In which section is the information available? (Reagents and Tools Table, Materials and Methods, Figures, Data Availability Section)
If collected and within the bounds of privacy constraints report on age, sex and gender or ethnicity for all study participants.	Not Applicable	

Core facilities	Information included in the manuscript?	In which section is the information available? (Reagents and Tools Table, Materials and Methods, Figures, Data Availability Section)
If your work benefited from core facilities, was their service mentioned in the acknowledgments section?	Not Applicable	

Design

Study protocol	Information included in the manuscript?	In which section is the information available? (Reagents and Tools Table, Materials and Methods, Figures, Data Availability Section)
If study protocol has been **pre-registered, provide DOI in the manuscript**. For clinical trials, provide the trial registration number **OR** cite DOI.	Not Applicable	
Report the **clinical trial registration number** (at ClinicalTrials.gov or equivalent), where applicable.	Not Applicable	

Laboratory protocol	Information included in the manuscript?	In which section is the information available? (Reagents and Tools Table, Materials and Methods, Figures, Data Availability Section)
Provide DOI OR other citation details if **external detailed step-by-step protocols** are available.	Not Applicable	

Experimental study design and statistics	Information included in the manuscript?	In which section is the information available? (Reagents and Tools Table, Materials and Methods, Figures, Data Availability Section)
Include a statement about **sample size** estimate even if no statistical methods were used.	Yes	Figures
Were any steps taken to minimize the effects of subjective bias when allocating animals/samples to treatment (e.g. **randomization procedure**)? If yes, have they been described?	Not Applicable	
Include a statement about **blinding** even if no blinding was done.	Not Applicable	
Describe **inclusion/exclusion criteria** if samples or animals were excluded from the analysis. Were the criteria pre-established?<br><br>If sample or data points were omitted from analysis, report if this was due to attrition or intentional exclusion and provide justification.	Not Applicable	
For every figure, are **statistical tests** justified as appropriate? Do the data meet the assumptions of the tests (e.g., normal distribution)? Describe any methods used to assess it. Is there an estimate of variation within each group of data? Is the variance similar between the groups that are being statistically compared?	Yes	Figures

Sample definition and in-laboratory replication	Information included in the manuscript?	In which section is the information available? (Reagents and Tools Table, Materials and Methods, Figures, Data Availability Section)
In the figure legends: state number of times the experiment was **replicated** in laboratory.	Yes	Figures
In the figure legends: define whether data describe **technical or biological replicates**.	Yes	Figures

Ethics

Ethics	Information included in the manuscript?	In which section is the information available? (Reagents and Tools Table, Materials and Methods, Figures, Data Availability Section)
Studies involving **human participants**: State details of **authority granting ethics approval** (IRB or equivalent committee(s), provide reference number for approval.	Not Applicable	
Studies involving **human participants**: Include a statement confirming that **informed consent** was obtained from all subjects and that the experiments conformed to the principles set out in the WMA Declaration of Helsinki and the Department of Health and Human Services Belmont Report.	Not Applicable	
Studies involving **human participants:** For publication of **patient photos**, include a statement confirming that consent to publish was obtained.	Not Applicable	
Studies involving experimental **animals**: State details of **authority granting ethics approval** (IRB or equivalent committee(s), provide reference number for approval. Include a statement of compliance with ethical regulations.	Not Applicable	
Studies involving **specimen and field samples:** State if relevant **permits** obtained, provide details of authority approving study; if none were required, explain why.	Not Applicable	

Dual Use Research of Concern (DURC)	Information included in the manuscript?	In which section is the information available? (Reagents and Tools Table, Materials and Methods, Figures, Data Availability Section)
Could your study fall under dual use research restrictions? Please check biosecurity documents and list of **select agents and toxins** (CDC): https://www.selectagents.gov/sat/list.htm	Not Applicable	
If you used a select agent, is the security level of the lab appropriate and reported in the manuscript?	Not Applicable	
If a study is subject to dual use research of concern regulations, is the name of the **authority granting approval and reference number** for the regulatory approval provided in the manuscript?	Not Applicable	

Reporting

The MDAR framework recommends adoption of discipline-specific guidelines, established and endorsed through community initiatives. Journals have their own policy about requiring specific guidelines and recommendations to complement MDAR.

Adherence to community standards	Information included in the manuscript?	In which section is the information available? (Reagents and Tools Table, Materials and Methods, Figures, Data Availability Section)
State if relevant guidelines or checklists (e.g., **ICMJE, MIBBI, ARRIVE, PRISMA**) have been followed or provided.	Not Applicable	
For **tumor marker prognostic studies,** we recommend that you follow the **REMARK** reporting guidelines (see link list at top right). See author guidelines, under 'Reporting Guidelines'. Please confirm you have followed these guidelines.	Not Applicable	
For **phase II and III randomized controlled trials**, please refer to the **CONSORT** flow diagram (see link list at top right) and submit the CONSORT checklist (see link list at top right) with your submission. See author guidelines, under 'Reporting Guidelines'. Please confirm you have submitted this list.	Not Applicable	

Data Availability

Data availability	Information included in the manuscript?	In which section is the information available? (Reagents and Tools Table, Materials and Methods, Figures, Data Availability Section)
Have **primary datasets** been deposited according to the journal's guidelines (see 'Data Deposition' section) and the respective accession numbers provided in the Data Availability Section?	Yes	Data Availability Section
Were **human clinical and genomic datasets** deposited in a public access-controlled repository in accordance to ethical obligations to the patients and to the applicable consent agreement?	Not Applicable	
Are **computational models** that are central and integral to a study available without restrictions in a machine-readable form? Were the relevant accession numbers or links provided?	Not Applicable	
If publicly available data were reused, provide the respective **data citations in the reference list**.	Not Applicable	
